# Evaluating the reliability, quality, and readability of AI-generated patient education on hallux valgus: a comparative study of large language models

**DOI:** 10.1186/s12911-026-03722-8

**Published:** 2026-07-30

**Authors:** Ali Can Koluman, Ebru Aloglu Ciftci, Mehmet Utku Ciftci, Başar Burak Cakmur, Nezih Ziroglu

**Affiliations:** 1 Department of Orthopaedics and Traumatology, Bakirkoy Dr. Sadi Konuk Training and Research Hospital, Istanbul, 34147 Turkey; 2 Division of Physiotherapy and Rehabilitation, Faculty of Health Sciences, Istanbul Okan University, Istanbul, 34959 Turkey; 3 Department of Physiotherapy and Rehabilitation, Institute of Graduate Education, Istinye University, Istanbul, 34010 Turkey; 4 Department of Orthopaedics and Traumatology, Sultan Abdülhamid Han Training and Research Hospital, Istanbul, 34668 Turkey; 5 Department of Orthopaedics and Traumatology, Beylikduzu State Hospital, Istanbul, 34500 Turkey; 6 Vocational School of Health Services, Department of Orthopedic Prosthetics and Orthotics, Acibadem Mehmet Ali Aydinlar University, Istanbul, 34638 Turkey; 7 Department of Orthopaedics and Traumatology, Acibadem University Atakent Hospital, Istanbul, 34303 Turkey

**Keywords:** Hallux valgus, Artificial intelligence, Large language models, Patient education, Readability, Health literacy

## Abstract

**Background:**

Patients with hallux valgus increasingly seek health information through consumer-facing artificial intelligence (AI)–driven patient education tools, particularly large language model–based conversational agents. Although these tools offer rapid and accessible responses, concerns remain regarding the reliability, usefulness, overall quality, and readability of AI-generated patient education materials. Evidence specifically evaluating AI-generated patient education for hallux valgus, a condition strongly influenced by patient expectations and treatment preferences, remains limited.

**Methods:**

This cross-sectional comparative study evaluated the performance of three large language models—ChatGPT-4o, Gemini-2.5-Flash, and DeepSeek-V3—in responding to 20 patient-centered questions related to hallux valgus. Questions were developed using AI-assisted question generation and publicly available Google Trends search patterns and categorized into four clinical domains. AI-generated responses were anonymized and independently assessed by three orthopaedic surgeons for reliability, usefulness, and overall quality using 7-point Likert-based reliability and usefulness scales and the Global Quality Scale (GQS). Readability was analyzed using six standardized indices. Inter-rater agreement and between-model comparisons were statistically evaluated.

**Results:**

Gemini-2.5-Flash demonstrated modestly higher overall reliability, particularly in questions related to etiology and clinical presentation. DeepSeek-V3 achieved higher usefulness scores in the long-term outcomes and quality-of-life domain and produced significantly more readable content, as reflected by higher Flesch Reading Ease scores and lower grade-level indices. In contrast, Gemini-2.5-Flash generated linguistically more complex responses requiring higher educational levels for comprehension. Overall usefulness and global quality scores did not differ significantly among models. Qualitative review also identified occasional examples of oversimplified or potentially misleading information. Despite these differences, several readability metrics exceeded recommended patient health-literacy thresholds.

**Conclusions:**

Contemporary AI-based conversational agents can provide patient-oriented information regarding hallux valgus with variable reliability and readability characteristics, although statistically significant differences were observed across models. A trade-off between factual accuracy and linguistic accessibility was observed. AI tools should therefore be regarded as adjuncts to, rather than replacements for, clinician-led patient education. Awareness of AI limitations and appropriate clinical guidance remain essential to ensure safe, accurate, and patient-centered information delivery.

**Clinical trial number:**

Not applicable.

**Supplementary Information:**

The online version contains supplementary material available at https://doi.org/10.1186/s12911-026-03722-8.

## Introduction

Hallux valgus (HV) is a common, progressive, and multifactorial deformity of the forefoot characterized by lateral deviation of the hallux and medial displacement of the first metatarsal bone [1, 2]. Clinically, HV is frequently associated with pain, functional limitation, difficulty with footwear, and deterioration in health-related quality of life, particularly among middle-aged and older individuals [3, 4]. The prevalence of HV in the general population has been reported to be approximately 19%, increasing to more than 23% in individuals aged 60 years and older, with a significantly higher prevalence among women compared to men [1, 5]. Owing to its high prevalence and chronic course, HV represents not only an orthopaedic condition but also a relevant public health issue.

Management of symptomatic HV encompasses a broad spectrum of interventions, ranging from conservative measures to surgical correction [6]. Traditionally, surgical success has been largely defined by radiographic parameters. However, patients’ expectations tend to focus more on pain relief, functional improvement, cosmetic appearance, and return to daily activities [7, 8]. Notably, previous studies have reported that approximately 25–33% of patients remain dissatisfied after HV surgery despite adequate radiographic correction and improvement in pain scores [9, 10]. These findings highlight a persistent discrepancy between surgeon-defined outcomes and patient-perceived success. Patient expectations have been identified as one of the strongest predictors of postoperative satisfaction, emphasizing the importance of effective preoperative education and expectation management [8, 11]. In this context, patients increasingly seek detailed information regarding disease progression, treatment options, postoperative recovery, and long-term outcomes, often outside the traditional healthcare system. Because many patients now obtain health information before clinical consultation, AI-generated content may shape expectations regarding conservative treatment, surgical indications, postoperative recovery, and likely outcomes before the surgeon-patient encounter.

In recent years, the use of artificial intelligence (AI), machine learning, and deep learning applications in orthopaedic surgery has expanded substantially [12, 13]. Within foot and ankle surgery, AI has been explored for tasks such as automated radiographic measurements and outcome prediction in conditions including HV [2, 13]. More recently, large language models (LLMs), such as AI-based conversational agents, have emerged as widely accessible tools for health information seeking. Patients increasingly use these platforms to obtain medical information due to their accessibility, conversational structure, and ability to rapidly generate individualized text-based responses [14, 15]. However, these advantages must be interpreted cautiously, as LLMs may generate inaccurate, incomplete, or fabricated medical information, commonly referred to as hallucinations [16]. In addition, patients may misinterpret AI-generated recommendations as individualized medical advice, potentially leading to inappropriate self-management or delayed clinical consultation.

Despite their growing popularity, important concerns remain regarding the reliability, scientific accuracy, and readability of AI-generated health information. International health communication guidelines recommend that patient education materials be written at approximately a sixth- to eighth-grade reading level to ensure accessibility across diverse populations. However, existing evaluations of LLM-generated medical content indicate that even high-performing models may struggle to consistently balance factual accuracy with appropriate readability [12, 17]. Content that is inaccurate, overly complex, or misleading may negatively influence patient understanding, expectations, and decision-making, particularly in conditions such as HV where treatment choices and outcomes are closely linked to patient preferences and expectations. Readability is particularly important in musculoskeletal patient education because treatment decisions often require patients to understand risks, benefits, alternatives, recovery timelines, and expected functional outcomes. Educational content written above the recommended literacy level may impair shared decision-making, reduce the quality of informed consent, and adversely affect adherence to postoperative rehabilitation or conservative treatment recommendations.

Although AI-generated patient education has been evaluated in several orthopaedic conditions, including meniscal injuries, rotator cuff disease, scoliosis, and spinal disorders, previous orthopaedic chatbot evaluation studies have primarily focused on sports injuries, spinal disorders, and general musculoskeletal conditions, whereas foot and ankle disorders remain comparatively underrepresented in the literature. HV represents a clinically relevant and preference-sensitive condition in which treatment decisions are strongly influenced by patient expectations, footwear concerns, cosmetic perception, and functional limitations. Therefore, evaluating AI-generated information specifically in HV is clinically relevant.

Therefore, the primary aim of this study was to compare the factual reliability of responses generated by three widely used LLMs to patient-centered questions regarding HV. Secondary aims were to evaluate response usefulness, overall quality, and readability using blinded orthopaedic specialist assessment and standardized multi-index readability analyses.

## Materials and methods

### Study design

This study was designed as a cross-sectional comparative analysis evaluating the performance of three large language models (LLMs)—ChatGPT-4o (OpenAI), Gemini-2.5-Flash (Google DeepMind), and DeepSeek-V3 (DeepSeek AI)—in providing patient-oriented information related to HV. The primary aim of the study was to assess the reliability, usefulness, overall quality, and readability of AI-generated responses to commonly asked patient questions regarding this condition. According to institutional policy, formal ethics committee approval was not required for this study because no human participants or patient data were involved. An official exemption/waiver confirmation was obtained from the institutional ethics committee. The study was designed as an exploratory comparative evaluation of contemporary LLM performance in patient-oriented orthopaedic information delivery.

### Question identification and development of the final question set

To generate an initial broad pool of potentially relevant patient-oriented questions, each AI chatbot was independently prompted using the same open-ended query regarding commonly asked questions about HV encountered in clinical practice. The exact prompt used for question generation was: “What are the most common questions patients ask about HV (bunions) during clinical consultation and online health-information seeking?”. This process resulted in an initial pool of 40 questions generated collectively by the three AI models. Duplicate and semantically overlapping questions were independently reviewed by two investigators and consolidated through consensus discussion. Questions considered clinically irrelevant, excessively vague, or rarely encountered in routine clinical practice were excluded based on expert consensus.

To improve ecological validity and better reflect real-world patient information-seeking behavior, the preliminary question pool was cross-referenced with publicly available Google Trends search patterns related to HV and bunion-related queries. Google Trends searches were performed using the terms “hallux valgus” and “bunion” between January 2024 and July 2025 using worldwide English-language search data. Frequently recurring patient-oriented search topics and commonly searched themes were qualitatively reviewed and considered during refinement and prioritization of the final question set. Following this multi-step refinement process, a final set of 20 patient-focused questions was established (Table 1).

Subsequently, three orthopaedic and traumatology surgeons independently reviewed the final question set and categorized each question according to its primary clinical focus into one of four thematic domains: etiology and clinical presentation, treatment options and surgical decision-making, postoperative course and functional recovery, and long-term outcomes and quality of life. The reviewers had 3, 4, and 7 years of post-residency clinical and academic experience in orthopaedic surgery, with subspecialty backgrounds in foot and ankle surgery, sports traumatology, and orthopaedic research. Disagreements regarding categorization were resolved through consensus discussion.

**Table 1:** Patient questions on hallux valgus

Category	No	Question
Etiology and Clinical Presentation	1	Why does hallux valgus occur?
	2	Is it genetic? If it runs in the family, can I get it too?
	3	Is shoe choice (high heel, narrow shoes) effective?
	4	Does flatfoot lead to hallux valgus?
	5	Is hallux valgus seen in children?
	6	Is pain, swelling and redness normal?
Treatment Options and Surgical Decision-Making	7	Is treatment without surgery possible?
	8	Do methods such as orthopedic insoles, splints, pads, exercises or taping work?
	9	In which cases is surgery necessary?
	10	What are the types of surgery?
	11	What should I do if my child has the onset of hallux valgus?
Postoperative Course and Functional Recovery	12	Is there pain after surgery and how long does it take to recover?
	13	When can I wear normal shoes after surgery?
	14	Will the surgery leave a scar?
	15	How long does it take to return to daily life and work?
	16	Will it recur after surgery?
Long-Term Outcomes and Quality of Life	17	Does hallux valgus worsen over time if left untreated?
	18	Does hallux valgus affect other fingers?
	19	Will it affect my walking or playing sports?
	20	Will losing weight reduce the symptoms?

### Response generation and blinded evaluation procedure

To minimize contextual memory effects and prevent cross-question contamination, each question was submitted individually to each AI model using separate browser sessions. All AI-generated responses were collected verbatim and subsequently anonymized. To reduce potential evaluator bias, a secondary researcher coded and de-identified all responses so that reviewers remained blinded to the AI model identity and response source throughout the evaluation process.

### Expert evaluation of AI-generated responses

Before formal scoring, reviewers participated in a pilot calibration session using sample AI-generated responses not included in the final analysis to improve scoring consistency and interpretation of evaluation criteria. All AI-generated responses were independently evaluated by three orthopaedic and traumatology specialists under blinded conditions using previously applied expert-based Likert scoring approaches and the validated Global Quality Scale (GQS). Reliability was assessed using a 7-point Likert scale, with higher scores reflecting greater scientific accuracy, internal consistency, and completeness of information. Usefulness was also evaluated using a 7-point Likert scale, taking into account clarity, comprehensibility, level of detail, and potential applicability for patient education. For reliability and usefulness assessments, scores of 1 indicated very poor performance with substantial inaccuracies or limited educational value, whereas scores of 7 reflected highly accurate, comprehensive, and clinically useful information (Supplementary Table S1). Overall quality was assessed using the 5-point Global Quality Scale (GQS), where lower scores indicated poor or incomplete content and higher scores reflected high-quality, comprehensive, and well-structured information suitable for patient education.

### Readability analysis

The readability of AI-generated responses was evaluated using six validated readability indices commonly applied in health communication research: the Flesch Reading Ease score, Flesch–Kincaid Grade Level, Gunning Fog Index, Coleman–Liau Index, Simple Measure of Gobbledygook, and Automated Readability Index. Higher Flesch Reading Ease scores indicate easier readability, whereas higher scores on the other indices correspond to increased reading difficulty and higher required educational levels. Prior to readability analysis, formatting artifacts, hyperlinks, bullet-point symbols, and duplicated spacing were removed without altering the semantic content of the responses. Readability outcomes were interpreted in relation to international health literacy recommendations, which suggest that patient education materials should be written at approximately a sixth- to eighth-grade reading level. All readability calculations were performed using the Readable.com online platform (United Kingdom), which provides standardized and widely accepted readability assessments for patient-oriented health information.

### Statistical analysis

All statistical analyses were performed using IBM SPSS Statistics version 27.0 (IBM Corp., Armonk, NY, USA). Descriptive statistics were reported as mean and standard deviation, median, and interquartile range, as appropriate. The normality of data distributions was assessed using the Shapiro–Wilk test.

Inter-rater reliability was evaluated using the intraclass correlation coefficient calculated with a two-way random-effects model and an absolute agreement definition. ICC values were interpreted according to established guidelines, with higher values indicating greater agreement among raters. To complement ICC analyses and examine overall agreement patterns among multiple raters, Fleiss’ kappa coefficients were calculated using R statistical software (version 4.5.0) and interpreted according to Landis and Koch criteria. Intraclass correlation coefficients (ICC) and 95% confidence intervals were calculated using a two‑way random‑effects, absolute‑agreement model following contemporary reporting recommendations.

Comparisons among AI models were conducted using the Kruskal–Wallis test, followed by Bonferroni-corrected post hoc analyses when appropriate. For readability indices that met normality assumptions, one-way analysis of variance was applied. Effect sizes were reported using eta squared, and a p-value of less than 0.05 was considered statistically significant.

To account for potential clustering of ratings derived from the same clinical questions, supplementary mixed-effects linear regression analyses were performed as sensitivity analyses, with question included as a random effect and AI model as a fixed effect. These analyses were conducted to evaluate the robustness of the primary findings after adjustment for within-question dependency.

Because the study was designed as an exploratory comparative analysis of AI-generated outputs rather than a hypothesis-testing clinical trial, a formal sample-size calculation was not performed. Kruskal–Wallis testing was selected as the primary analytical approach given the ordinal nature of Likert-scale ratings and non-normal score distributions confirmed by the Shapiro–Wilk test.

### Model versioning and access dates

We accessed ChatGPT‑4o (OpenAI), Gemini‑2.5‑Flash (Google), and DeepSeek‑V3 (DeepSeek AI) between July and August 2025. All prompts were entered via the official web user interfaces; browsing or plug‑ins were disabled. No regenerative sampling beyond the first complete response was allowed unless a model produced an explicit refusal. Default platform settings were used for all models, and no manual adjustment of generation parameters was performed.

## Results

### Inter-rater agreement

Inter-rater reliability analyses demonstrated a high level of agreement among the three orthopaedic surgeons across all AI models and evaluation domains (Table 2). Intraclass correlation coefficient (ICC) values ranged from 0.787 to 0.981, indicating good to excellent reliability for all assessed parameters.

Gemini-2.5-Flash showed the highest inter-rater agreement for reliability assessments (ICC = 0.869), whereas DeepSeek-V3 demonstrated the highest agreement for usefulness (ICC = 0.979) and overall quality as measured by the Global Quality Scale (GQS) (ICC = 0.981).

Consistent with ICC findings, Fleiss’ kappa analyses revealed moderate to almost perfect agreement across all AI models and evaluation criteria (κ = 0.587–0.911; all *p* < 0.001). Gemini exhibited the highest agreement for reliability (κ = 0.866) and GQS (κ = 0.911), while DeepSeek achieved the highest agreement for usefulness ratings (κ = 0.790) (Table 2). These findings support the overall consistency of expert evaluations across AI models and assessment domains.

**Table 2:** Inter-rater reliability and agreement of AI models across evaluation criteria assessed by ICC and fleiss’ kappa

AI Models	Evaluation Criteria	ICC (95% CI)	Interpretation (ICC)	Fleiss’ kappa [z]	Interpretation (κ)
ChatGPT-4o	**Reliability**	0.809 (0.602–0.918)	Good	0.839 [8.77]	Almost perfect
	**Usefulness**	0.949 (0.894–0.978)	Excellent	0.587 [6.45]	Moderate
	**GQS**	0.962 (0.921–0.984)	Excellent	0.872 [9.5]	Almost perfect
Gemini-2.5-Flash	**Reliability**	0.869 (0.722–0.944)	Excellent	0.886 [8.96]	Almost perfect
	**Usefulness**	0.913 (0.819–0.963)	Excellent	0.713 [7.36]	Substantial
	**GQS**	0.961 (0.919–0.983)	Excellent	0.911 [8.43]	Almost perfect
Deepseek-V3	**Reliability**	0.787 (0.559–0.908)	Good	0.847 [9.13]	Almost perfect
	**Usefulness**	0.979 (0.957–0.991)	Excellent	0.790 [9.21]	Substantial
	**GQS**	0.981 (0.961–0.992)	Excellent	0.856 [8.7]	Almost perfect

GQS, Global quality score; ICC, intraclass correlation coefficient; 95% CI: 95% Confidence Interval; κ, Fleiss’ kappa; z, Z-statistic for testing the statistical significance of Fleiss’ kappa

### Performance in the etiology and clinical presentation domain

In the etiology and clinical presentation domain, statistically significant differences were observed among AI models with respect to reliability scores (η² = 0.432, *p* = 0.014) (Table 3). Gemini-2.5-Flash demonstrated statistically higher reliability scores (5.50 ± 0.45) compared with DeepSeek-V3 (4.55 ± 0.50; *p* = 0.036), although the absolute difference in scores was modest. No significant differences were identified between models for usefulness (η² = 0.178, *p* = 0.097) or overall quality (GQS) (η² = 0.032, *p* = 0.466).

### Performance in the treatment options and surgical decision-making domain

In the treatment options and surgical decision-making domain, a statistically significant overall difference in reliability scores was detected among AI models (η² = 0.337, *p* = 0.049). However, post hoc pairwise comparisons did not reveal significant differences between individual models. No statistically significant differences were observed for usefulness (η² = 0.103, *p* = 0.198) or GQS scores (η² = 0.114, *p* = 0.730) across models (Table 3).

### Performance in the postoperative course and functional recovery domain

For questions related to postoperative course and functional recovery, no statistically significant differences were identified among AI models in terms of reliability (η² = 0.215, *p* = 0.101), usefulness (η² = 0.007, *p* = 0.353), or overall quality (GQS) (η² = 0.139, *p* = 0.846) (Table 3). No statistically significant differences were identified among AI models across any evaluated parameter within this domain.

**Table 3:** Reliability, usefulness, and quality (GQS) scores of AI-generated responses on hallux valgus

Criterion	Domain	AI Models	Mean ± SD	Median (IQR)	η²	Test Statistic	*p*	Significant pairwise differences
Reliability	Etiology and Clinical Presentation	ChatGPT-4o	4.94 ± 0.32	5.00 (0.25)	0.432	8.473	**0.014**	Gemini> Deepseek (*p* = 0.036)
		Gemini	5.50 ± 0.45	5.50 (1.00)				
		DeepSeek	4.55 ± 0.50	4.66 (1.00)				
	Treatment Options and Surgical Decision-Making	ChatGPT-4o	5.00 ± 0.62	5.00 (1.00)	0.337	6.049	**0.049**	ns (no sig. Difference)
		Gemini	5.73 ± 0.43	6.00 (0.67)				
		DeepSeek	4.86 ± 0.67	5.00 (0.67)				
	Postoperative Course and Functional Recovery	ChatGPT-4o	4.93 ± 0.59	5.00 (0.83)	0.215	4.584	0.101	ns
		Gemini	5.73 ± 0.43	6.00 (0.67)				
		DeepSeek	5.13 ± 0.64	4.66 (1.17)				
	Long-Term Outcomes and Quality of Life	ChatGPT-4o	5.41 ± 0.50	5.33 (0.92)	0.327	4.941	0.085	ns
		Gemini	6.00 ± 0.00	6.00 (0.00)				
		DeepSeek	5.33 ± 0.47	5.16 (0.83)				
	All Questions	ChatGPT-4o	5.05 ± 0.51	5.00 (0.33)	0.327	20.640	**< 0.001**	Gemini> ChatGPT (*p* < 0.001); Gemini> Deepseek (*p* < 0.001)
		Gemini	5.71 ± 0.40	6.00 (0.58)				
		DeepSeek	4.93 ± 0.57	5.00 (0.58)				
Usefulness	Etiology and Clinical Presentation	ChatGPT-4o	5.16 ± 0.40	5.00 (0.25)	0.178	4.673	0.097	ns
		Gemini	5.77 ± 0.40	6.00 (0.50)				
		DeepSeek	5.38 ± 0.49	5.16 (1.00)				
	Treatment Options and Surgical Decision-Making	ChatGPT-4o	5.60 ± 0.54	6.00 (1.00)	0.103	3.240	0.198	ns
		Gemini	5.46 ± 0.76	6.00 (1.33)				
		DeepSeek	4.86 ± 0.69	4.66 (1.17)				
	Postoperative Course and Functional Recovery	ChatGPT-4o	5.33 ± 0.84	5.66 (1.50)	0.007	2.080	0.353	ns
		Gemini	5.53 ± 0.73	6.00 (1.17)				
		DeepSeek	6.00 ± 1.22	6.00 (2.00)				
	Long-Term Outcomes and Quality of Life	ChatGPT-4o	5.75 ± 0.31	5.83 (0.58)	0.709	8.379	**0.015**	Deepseek> ChatGPT (*p* = 0.039); Deepseek> Gemini (*p* = 0.033)
		Gemini	5.83 ± 0.33	6.00 (0.50)				
		DeepSeek	7.00 ± 0.00	7.00 (0.00)				
	All Questions	ChatGPT-4o	5.43 ± 0.57	5.50 (1.00)	0.002	1.873	0.392	ns
		Gemini	5.65 ± 0.56	6.00 (0.67)				
		DeepSeek	5.73 ± 1.03	6.00 (2.00)				
GQS	Etiology and Clinical Presentation	ChatGPT-4o	4.38 ± 0.44	4.33 (0.75	0.032	1.526	0.466	ns
		Gemini	4.16 ± 0.27	4.00 (0.42)				
		DeepSeek	4.00 ± 0.55	4.00 (0.67)				
	Treatment Options and Surgical Decision-Making	ChatGPT-4o	4.00 ± 1.00	4.00 (2.00)	0.114	0.630	0.730	ns
		Gemini	4.40 ± 0.89	5.00 (1.50)				
		DeepSeek	4.00 ± 1.00	4.00 (2.00)				
	Postoperative Course and Functional Recovery	ChatGPT-4o	3.66 ± 0.84	3.33 (1.50)	0.139	0.334	0.846	ns
		Gemini	3.66 ± 0.62	4.00 (1.17)				
		DeepSeek	4.00 ± 1.00	4.00 (2.00)				
	Long-Term Outcomes and Quality of Life	ChatGPT-4o	4.66 ± 0.47	4.83 (0.83)	0.206	0.143	0.931	ns
		Gemini	4.50 ± 0.57	4.50 (1.00)				
		DeepSeek	4.50 ± 0.57	4.50 (1.00)				
	All Questions	ChatGPT-4o	4.16 ± 0.76	4.00 (1.50)	0.034	0.072	0.965	ns
		Gemini	4.16 ± 0.65	4.00 (0.92)				
		DeepSeek	4.10 ± 0.77	4.00 (1.75)				

Kruskal-Wallis test; IQR, interquartile range; SD, standard deviation; Test statistic, H values from Kruskal-Wallis Test; η2, Eta squared; Sections highlighted in bold indicate statistical significance (p<0.05)

### Performance in the long-term outcomes and quality of life domain

**Fig. 1:**
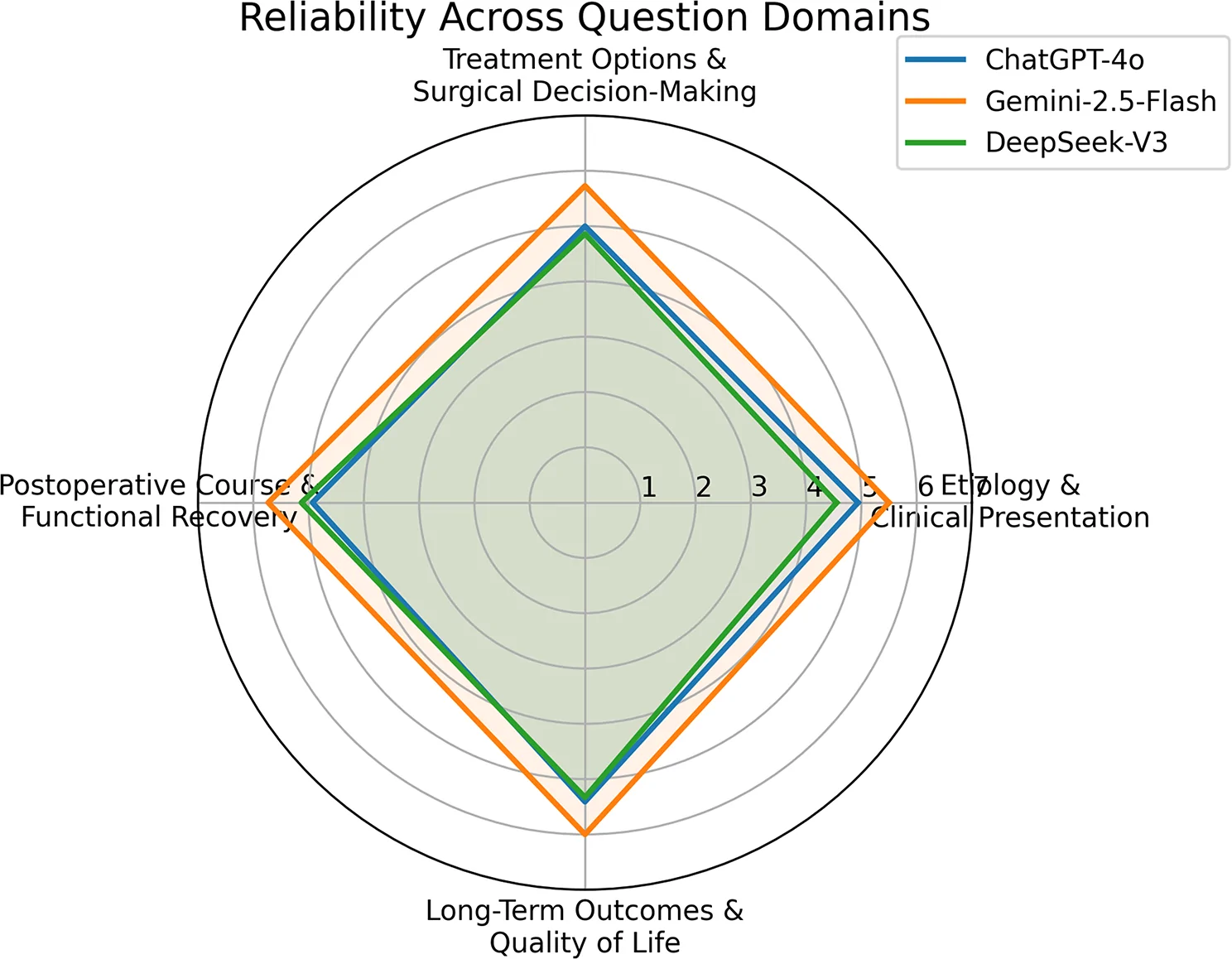
Reliability scores of ChatGPT-4o, Gemini-2.5-Flash, and DeepSeek-V3 across the four clinical domains related to HV (etiology and clinical presentation, treatment options and surgical decision-making, postoperative course and functional recovery, and long-term outcomes and quality of life). Gemini-2.5-Flash demonstrated higher reliability across most domains, whereas differences among models were less pronounced in postoperative and long-term outcome–related questions

In the long-term outcomes and quality of life domain, reliability (η² = 0.327, *p* = 0.085) and GQS scores (η² = 0.206, *p* = 0.931) did not differ significantly among AI models. In contrast, usefulness scores showed a statistically significant difference (η² = 0.709, *p* = 0.015). DeepSeek-V3 demonstrated statistically higher usefulness ratings compared with both ChatGPT-4o (*p* = 0.039) and Gemini-2.5-Flash (*p* = 0.033) (Table 3; Fig. 1.).

### Overall performance across all question domains

**Fig. 2:**
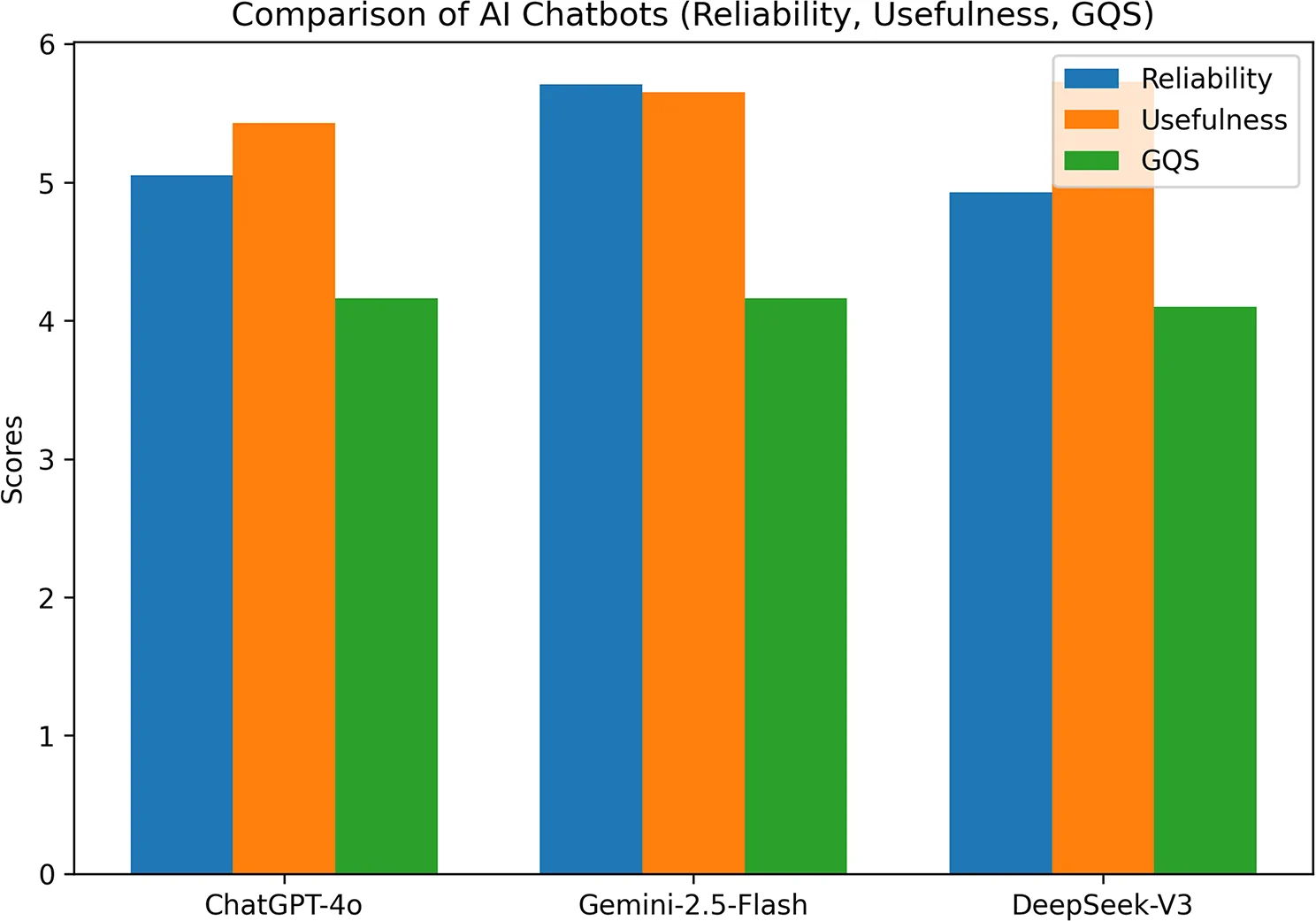
Comparison of overall reliability, usefulness, and Global Quality Scale (GQS) scores among ChatGPT-4o, Gemini-2.5-Flash, and DeepSeek-V3 across all patient-centered questions related to HV. Gemini-2.5-Flash demonstrated significantly higher overall reliability, whereas no significant differences were observed among models in terms of usefulness or global quality

When all question domains were analyzed collectively, significant differences were observed among AI models in terms of reliability (η² = 0.327, *p* < 0.001). Post hoc analyses revealed that Gemini-2.5-Flash demonstrated modestly higher overall reliability compared with both ChatGPT-4o (*p* < 0.001) and DeepSeek-V3 (*p* < 0.001), although differences across models remained within a relatively narrow scoring range (Fig. 2.). No statistically significant differences were observed among models for overall usefulness (η² = 0.002, *p* = 0.392) or GQS scores (η² = 0.034, *p* = 0.965) (Table 3).

Sensitivity analyses using mixed-effects models accounting for clustering at the question level demonstrated findings broadly consistent with the primary analyses. Gemini remained associated with modestly higher reliability scores compared with ChatGPT (β = 0.667, *p* < 0.001), whereas no significant differences were observed for overall GQS scores between models. For usefulness, DeepSeek demonstrated modestly higher scores compared with ChatGPT (β = 0.300, *p* = 0.014), while the comparison between Gemini and ChatGPT was no longer statistically significant. (Supplementary Table S2.)

### Comparison of readability metrics

Comparative analysis of readability metrics revealed statistically significant differences among AI models across all evaluated indices (all *p* < 0.001) (Table 4). DeepSeek-V3 achieved the highest Flesch Reading Ease (FRE) scores, indicating easier readability and easier comprehension compared with the other models (*p* < 0.05). Conversely, Gemini-2.5-Flash consistently produced content with higher Flesch-Kincaid Grade Level (FKGL), Gunning Fog Index (GFI), Coleman-Liau Index (CLI), SMOG, and Automated Readability Index (ARI) scores, reflecting greater linguistic complexity and higher required reading levels (*p* < 0.05). Despite these differences, several readability metrics indicated that the generated responses frequently exceeded the commonly recommended sixth- to eighth-grade reading level for patient education materials. Mean response length also differed significantly across models, with Gemini-2.5-Flash producing the longest responses (mean 301.9 ± 70.1 words), followed by DeepSeek-V3 (294.6 ± 62.7 words) and ChatGPT-4o (178.7 ± 49.1 words).

**Table 4:** Comparison of readability scores among ai models based on different readability metrics and response length

Readability Metrics	AI Models	Mean ± SD	Median [IQR]	η²	*p*-value (Pairwise)
FRE	ChatGPT-4o	49.77 ± 8.57	49.50 [14]	0.312	Deepseek > ChatGPT (*p* = 0.023) Deepseek > Gemini (*p* < 0.001)
	Gemini	43.57 ± 8.22	44.96 [11.79]		
	Deepseek	57.19 ± 8.65	55.48 [12.99]		
FKGL	ChatGPT-4o	9.80 ± 1.32	9.65 [1.50]	0.599	All comparisons *p* < 0.001
	Gemini	11.61 ± 1.29	11.55 [2.01]		
	Deepseek	7.78 ± 1.31	8.21 [2.33]		
GFI	ChatGPT-4o	12.01 ± 1.92	12.07 [2.24]	0.447	Gemini > ChatGPT (*p* = 0.009) Chatgpt > Deepseek (*p* = 0.002) Gemini > Deepseek (*p* < 0.001)
	Gemini	13.69 ± 1.77	13.45 [2.64]		
	Deepseek	10.01 ± 1.40	10.56 [2.38]		
CLI	ChatGPT-4o	10.85 ± 1.24	10.88 [1.84]	0.522	Gemini > ChatGPT (*p* = 0.002) Chatgpt > Deepseek (*p* < 0.001) Gemini > Deepseek (*p* < 0.001)
	Gemini	12.49 ± 1.29	12.17 [1.85]		
	Deepseek	8.98 ± 1.64	9.22 [3.17]		
SMOG	ChatGPT-4o	11.95 ± 1.05	11.98 [1.46]	0.615	All comparisons *p* < 0.001
	Gemini	13.31 ± 1.03	13.35 [1.60]		
	Deepseek	10.10 ± 1.11	10.36 [1.72]		
ARI	ChatGPT-4o	8.64 ± 1.30	8.30 [1.97]	0.685	All comparisons *p* < 0.001
	Gemini	11.48 ± 1.33	11.51 [2.25]		
	Deepseek	6.06 ± 1.90	5.86 [2.90]		
Word count (per response)	ChatGPT-4o	178.7 ± 49.1	179 [68]	0.281	Gemini > ChatGPT (*p* < 0.001) DeepSeek > ChatGPT (*p* < 0.001)
	Gemini	301.9 ± 70.1	302 [112]		
	Deepseek	294.6 ± 62.7	305 [94]		

All overall comparisons were performed using one-way ANOVA, with all omnibus p-values < 0.001. Pairwise comparisons were conducted using the Bonferroni post hoc test, and adjusted p-values are reported as *p* (pairwise); η2, Eta squared; FRE, *Flesch Reading Ease;* FKGL, *Flesch-Kincaid Grade Level*; GFI, *Gunning Fog Index*; CLI, *Coleman-Liau Index*, SMOG, *Simple Measure of Gobbledygook*; ARI, *Automated Readability Index* The sections highlighted in bold are statistically significant (*p* < 0.05)

### Representative examples of potentially misleading information

Qualitative review of AI-generated responses identified occasional examples of potentially misleading or oversimplified information. Common issues included overly optimistic descriptions of conservative treatment efficacy, limited discussion of recurrence risk, and inconsistent descriptions of postoperative recovery timelines. Representative examples are provided in Supplementary Table S3. And also, a summary of the principal findings and their clinical implications is provided in Supplementary Table S4.

## Discussion

The present study provides a comprehensive evaluation of AI-generated patient education content on HV by comparing three widely used large language models with respect to reliability, usefulness, overall quality, and readability. The principal findings of this study indicate that although all evaluated models were capable of generating patient-oriented information with moderate overall reliability and variable readability characteristics, important differences exist in terms of reliability and readability, highlighting that no single model consistently demonstrated a balanced combination of factual reliability and readability.

One of the most notable findings was the high inter-rater agreement among orthopaedic specialists across all evaluation domains, as demonstrated by excellent ICC and Fleiss’ kappa values. This finding supports the overall consistency of the expert-based evaluation framework and suggests that expert-based assessment of AI-generated medical content can be performed with a high degree of consistency. Similar levels of inter-rater reliability have been reported in previous studies evaluating AI-generated patient education materials in orthopaedics and other medical fields, reinforcing the validity of expert-based scoring systems in this context.

When individual content domains were examined, Gemini-2.5-Flash demonstrated higher reliability scores, particularly in the etiology and clinical presentation domain and across all questions combined. This finding aligns with recent literature suggesting that newer-generation large language models may perform better in conveying foundational medical knowledge with higher internal consistency [18, 19]. Although statistically significant differences in reliability were observed among the models, the absolute magnitude of these differences was relatively modest (approximately 0.6–0.8 points on a 7-point scale). Therefore, the practical clinical impact of these differences on patient understanding and decision-making should be interpreted cautiously. In contrast, DeepSeek-V3 achieved significantly higher usefulness scores in the long-term outcomes and quality of life domain. This observation suggests that some models may generate responses perceived as more useful for broader patient-centered concerns rather than technical or pathophysiological aspects of disease [12, 17, 18]. Such variability across domains emphasizes that the performance of AI chatbots is context-dependent and cannot be generalized across all aspects of patient education.

Importantly, overall usefulness and global quality scores did not differ significantly among models when all domains were considered collectively. This finding is consistent with prior comparative studies in musculoskeletal conditions, which have demonstrated that different AI models may reach similar perceived quality levels despite underlying differences in accuracy, depth, or presentation style [18–20]. From a clinical perspective, this reinforces the notion that AI-generated information may appear subjectively satisfactory to users despite differences in scientific reliability and content structure [21–24]. 

Readability analysis revealed the most pronounced differences among the evaluated models. DeepSeek-V3 produced content that was significantly easier to read, as reflected by higher Flesch Reading Ease scores and lower grade-level indices, whereas Gemini-2.5-Flash generated linguistically more complex content requiring higher educational levels for comprehension. These findings are highly relevant in the context of patient education, as international health literacy guidelines consistently recommend that educational materials be written at approximately a sixth- to eighth-grade reading level to ensure adequate patient understanding [25–27]. Previous studies evaluating AI-generated health information have similarly reported that increased factual detail often comes at the expense of readability, underscoring a persistent trade-off between accuracy and accessibility in AI-generated medical content [28–32]. Nevertheless, several readability indices across all evaluated models exceeded the commonly recommended sixth- to eighth-grade reading level for patient education materials, suggesting that readability challenges remain even in the most accessible responses. Notably, even the most readable model frequently exceeded the recommended sixth- to eighth-grade readability threshold for patient education materials. Because hallux valgus predominantly affects middle-aged and older adults, elevated readability levels may disproportionately limit accessibility among populations with lower health literacy, who constitute a large proportion of patients with this condition.

The clinical implications of these findings are particularly important in HV, where patient expectations play a critical role in treatment satisfaction and decision-making. Misinterpretation or oversimplification of information regarding disease progression, surgical indications, or postoperative recovery may contribute to unrealistic expectations and subsequent dissatisfaction, even in cases with objectively successful outcomes [7, 33]. In this context, AI-based conversational agents should be viewed as adjunctive tools that complement, rather than replace, clinician-led patient education [32, 34]. In clinical practice, AI-generated content may be useful as a supplementary educational resource or as a starting point for patient questions, provided that information is subsequently clarified and verified during clinician-patient communication. Clinicians should remain aware of the limitations of AI-generated content and guide patients toward reliable, comprehensible information sources [34–36]. Clinicians may also help patients formulate more focused and clinically appropriate prompts and encourage verification of AI-generated information through physician-reviewed or institutional educational resources. From a health informatics perspective, these findings highlight the need for governance frameworks that balance accuracy, accessibility, and ethical deployment of generative AI in patient education. Qualitative review of the generated responses also identified occasional examples of oversimplified or potentially misleading information, including overly optimistic descriptions of conservative treatment efficacy, limited discussion of recurrence risk, and inconsistent postoperative recovery expectations. Current evidence suggests that nonoperative modalities such as orthotics, splints, and footwear modifications may improve symptoms and footwear tolerance, although they do not reliably prevent or reverse HV deformity progression [37]. These findings are important because AI-generated responses occasionally appeared to overstate the corrective potential of conservative treatment modalities. Therefore, overly optimistic AI-generated descriptions of conservative treatment efficacy may contribute to unrealistic patient expectations and misunderstanding regarding the goals and limitations of nonoperative management.

### Limitations and future directions

Several limitations of this study should be acknowledged. First, the evaluation focused on English-language responses, and findings may not be generalizable to non-English-speaking populations. Second, AI models are continuously updated, and their performance may evolve over time. Third, patient perceptions were not directly assessed, and future studies incorporating patient-reported understanding and satisfaction would provide additional insight. AI-generated responses may vary according to prompt phrasing, platform updates, and temporal model modifications, which may limit full reproducibility of findings. In addition, the statistical analyses may not have fully accounted for potential within-question dependency and repeated-measures correlations across evaluators and AI models. The initial use of AI-assisted question generation may also have influenced question selection despite subsequent expert refinement and comparison with public search trends. Finally, although HV represents a common and clinically relevant model condition, findings may not be directly applicable to other foot and ankle disorders with different educational demands.

Future research should focus on refining AI-generated medical content through clinician-guided optimization, integrating health literacy principles into model development, and exploring hybrid approaches that combine AI efficiency with human oversight. Such strategies may enhance the safety, accuracy, and accessibility of AI-based patient education and support their responsible integration into digital health communication. In addition, ethical concerns related to misinformation, hallucinated content, lack of source transparency, and potential overreliance on non-validated AI-generated recommendations should be considered when integrating generative AI tools into patient education pathways. Although the primary analyses used non-parametric group comparisons, supplementary mixed-effects sensitivity analyses demonstrated similar overall findings after accounting for within-question clustering.

Despite these limitations, this study has several notable strengths. First, it employs a blinded, expert-based evaluation framework with high inter-rater agreement, supporting the consistency of the findings. Second, the use of a patient-centered question set informed by real-world search trends was intended to improve the clinical relevance and ecological validity. Third, the simultaneous assessment of reliability, usefulness, overall quality, and multiple validated readability indices provides a comprehensive and multidimensional evaluation of AI-generated patient education content. Finally, by focusing on HV, a common and clinically relevant condition, this study offers condition-specific insights that may inform the cautious and clinically supervised integration of AI-based tools into patient education.

## Conclusions

This study demonstrates that contemporary large language models are capable of providing patient-oriented information with variable reliability and readability characteristics on HV; however, statistically significant differences were observed in reliability and readability across models. While some AI systems demonstrated higher reliability scores, others provide more accessible and readable content, suggesting a trade-off between accuracy and comprehensibility. Importantly, readability levels across several models frequently exceeded recommended health-literacy thresholds for patient education materials. Given the critical role of patient education and expectation management in HV, AI-based conversational agents should be regarded as supportive tools rather than substitutes for clinician-led communication, particularly for preliminary education and reinforcement of clinician-provided information. Awareness of the limitations of AI-generated content and appropriate clinical guidance remain essential to ensure safe, accurate, and patient-centered information delivery. Future efforts should focus on integrating health literacy principles and clinician oversight to optimize the safe and clinically responsible integration of AI into patient education pathways.

## Supplementary Information

Below is the link to the electronic supplementary material.

Supplementary Material 1

Supplementary Material 2

## Data Availability

The datasets generated and analyzed during the current study, including expert rating scores and readability metrics, are available from the corresponding author upon reasonable request.

## Abbreviations

GQS: Global quality scale
ICC: Intraclass correlation coefficient
CI: Confidence interval
IQR: Interquartile range
SD: Standard deviation
FRE: Flesch reading ease
FKGL: Flesch-kincaid grade level
GFI: Gunning fog index
CLI: Coleman-Liau index
SMOG: Simple measure of gobbledygook
ARI: Automated readability index

## References

[1] Ettinger S, Spindler FT, Marschall U, Polzer H, Stukenborg-Colsman C, Baumbach SF. Hallux valgus: Prevalence and treatment options. Dtsch Ärztebl Int. 2025. 10.3238/arztebl.m2025.0068. PMC12539686 40332014

[2] Kwolek K, Gądek A, Kwolek K, Lechowska-Liszka A, Malczak M, Liszka H. Artificial intelligence-based diagnosis of hallux valgus interphalangeus using anteroposterior foot radiographs. World J Orthop. 2025;16. 10.5312/wjo.v16.i6.103832. PMC12179893 40547248

[3] Tengman E, Brodén C, Söderpalm A-C, Cöster MC. Patient-Reported Outcomes Before and After Hallux Valgus Surgery: 2-Year Results From a National Registry Study. Foot Ankle Orthop. 2025;10:24730114251351634. 10.1177/24730114251351634. PMC12301585 40727083

[4] Imran B, Jones S, Middleton RG. Hallux valgus management: an update based on National Institute for Health and Care Excellence (NICE) guidelines. Cureus. 2025;17(11):e96642. 10.7759/cureus.96642. PMC12699283 41393558

[5] Cai Y, Song Y, He M, He W, Zhong X, Wen H, et al. Global prevalence and incidence of hallux valgus: a systematic review and meta-analysis. J Foot Ankle Res. 2023;16:63. 10.1186/s13047-023-00661-9. PMC10510234 37726760

[6] Abdulsamad AM, Alfarag AH, Alsaqer O, Alqahtani AH, Alhawas AY, Alassaf TK, et al. Comparison of Scarf With and Without Akin Osteotomy for Hallux Valgus. Cureus. 2025. 10.7759/cureus.96498. PMC12694752 41384199

[7] Albright RH, Arp J, Bhatt Z, Weil L, Longacre M, Fleischer AE. Patient expectations in hallux valgus surgery: A qualitative analysis. J Foot Ankle Surg. 2025;64:120–5. 10.1053/j.jfas.2024.10.005. PMC11925686 39561851

[8] Kömür BH, Bahar H, Güler M. Halluks valgus hastalığının cerrahi tedavisinde hasta beklentisi. TOTBİD Derg. 2024;23:295–8. 10.5578/totbid.dergisi.2024.42.

[9] Albright R, Klein E, Baker J, Sorensen M, Weil L, Fleischer A. Are Radiographs Associated With Patient Satisfaction After Scarf Bunionectomy? J Foot Ankle Surg. 2023;62:2–6. 10.1053/j.jfas.2022.05.001. 35705454

[10] Ferrari J, Higgins JP, Prior TD. Interventions for treating hallux valgus (abductovalgus) and bunions. editor. Cochrane Database of Systematic Reviews. Chichester, UK: John Wiley & Sons, Ltd; 2004. 10.1002/14651858.CD000964.pub2. The Cochrane Collaborationp. CD000964.pub2. 14973960

[11] Matthews M, Klein E, Youssef A, Weil L, Sorensen M, Weil LS, et al. Correlation of Radiographic Measurements With Patient-Centered Outcomes in Hallux Valgus Surgery. Foot Ankle Int. 2018;39:1416–22. 10.1177/1071100718790255. 30136598

[12] Çakmur BB, Koluman AC, Çiftçi MU, Aloğlu Çiftçi E, Ziroğlu N. Gemini 1.5 Flash provides the most reliable content while ChatGPT-4o offers the highest readability for patient education on meniscal tears. Knee Surg Sports Traumatol Arthrosc. 2025. 10.1002/ksa.70247. :ksa.70247. PMC12948341 41451752

[13] Gupta P, Kingston KA, O’Malley M, Williams RJ, Ramkumar PN. Advancements in Artificial Intelligence for Foot and Ankle Surgery: A Systematic Review. Foot Ankle Orthop. 2023;8:24730114221151079. 10.1177/24730114221151079. PMC9929923 36817020

[14] Wang X, Shi J, Kong H. Online Health Information Seeking: A Review and Meta-Analysis. Health Commun. 2021;36:1163–75. 10.1080/10410236.2020.1748829. 32290679

[15] Wardle C, Urbani S, Wang E. Evolving Health Information–Seeking Behavior in the Context of Google AI Overviews, ChatGPT, and Alexa: Interview Study Using the Think-Aloud Protocol. J Med Internet Res. 2025;27:e79961. 10.2196/79961. PMC12541266 41055948

[16] Abd-alrazaq A, AlSaad R, Alhuwail D, Ahmed A, Healy PM, Latifi S, et al. Large Language Models in Medical Education: Opportunities, Challenges, and Future Directions. JMIR Med Educ. 2023;9:e48291. 10.2196/48291. PMC10273039 37261894

[17] Koluman AC, Çiftçi MU, Çiftçi EA, Çakmur BB, Ziroğlu N. Balancing Accuracy and Readability: Comparative Evaluation of AI Chatbots for Patient Education on Rotator Cuff Tears. Healthcare. 2025;13:2670. 10.3390/healthcare13212670. PMC12609729 41228037

[18] Uz C, Umay E. Dr ChatGPT : Is it a reliable and useful source for common rheumatic diseases? Int J Rheum Dis. 2023;26:1343–9. 10.1111/1756-185X.14749. 37218530

[19] Warren E, Hurley ET, Park CN, Crook BS, Lorentz S, Levin JM, et al. Evaluation of information from artificial intelligence on rotator cuff repair surgery. JSES Int. 2024;8:53–7. 10.1016/j.jseint.2023.09.009. PMC10837709 38312282

[20] Çıracıoğlu AM, Dal Erdoğan S. Evaluation of the reliability, usefulness, quality and readability of ChatGPT’s responses on Scoliosis. Eur J Orthop Surg Traumatol. 2025;35:123. 10.1007/s00590-025-04198-4. 40100428

[21] Bender EM, Gebru T, McMillan-Major A, Shmitchell S. On the dangers of stochastic parrots: can language models be too big? In: Proceedings of the 2021 ACM Conference on Fairness, Accountability, and Transparency. Virtual Event, Canada: Association for Computing Machinery; 2021. p. 610-623. 10.1145/3442188.3445922.

[22] Hernandez F, Guizar R, Avetisian H, Abdou MA, Karakash WJ, Ton A, et al. Evaluating the accuracy and readability of ChatGPT in addressing patient queries on adult spinal deformity surgery. Glob Spine J. 2025;21925682251360655. 10.1177/21925682251360655. PMC12254131 40643892

[23] Ayers JW, Poliak A, Dredze M, Leas EC, Zhu Z, Kelley JB, et al. Comparing Physician and Artificial Intelligence Chatbot Responses to Patient Questions Posted to a Public Social Media Forum. JAMA Intern Med. 2023;183:589. 10.1001/jamainternmed.2023.1838. PMC10148230 37115527

[24] Koluman AC, Yiğitbay A, Aloğlu Çiftçi E, Çiftçi MU, Ziroğlu N, Kural C. Assessing the accuracy, safety, and clinical utility of AI-generated rehabilitation guidance for Bankart lesions: a blinded comparative evaluation with expert protocols. J Med Palliat Care. 2026;7:274–81. 10.47582/jompac.1884791.

[25] Hartnett DA, Philips AP, Daniels AH, Blankenhorn BD. Readability of Online Foot and Ankle Surgery Patient Education Materials. Foot Ankle Spec. 2025;18:9–18. 10.1177/19386400221116463. 35934974

[26] Weiss BD. Health literacy and patient safety: help patients understand. Chicago (IL): American Medical Association and AMA Foundation; 2007.

[27] Jackson DN, Trivedi N, Baur C. Re-Prioritizing Digital Health and Health Literacy in *Healthy People 2030* to Affect Health Equity. Health Commun. 2021;36:1155–62. 10.1080/10410236.2020.1748828. 32354233

[28] Nasra M, Jaffri R, Pavlin-Premrl D, Kok HK, Khabaza A, Barras C, et al. Can artificial intelligence improve patient educational material readability? A systematic review and narrative synthesis. Intern Med J. 2025;55:20–34. 10.1111/imj.16607. 39720869

[29] Arcot D, Pondicherry N, Chakraborty S. Evaluating the quality of health information: comparison of human and artificial intelligence. Neurogastroenterol Motil. 2025;e70164. 10.1111/nmo.70164. PMC13244121 40993963

[30] Wang L, Li J, Zhuang B, Huang S, Fang M, Wang C, et al. Accuracy of Large Language Models When Answering Clinical Research Questions: Systematic Review and Network Meta-Analysis. J Med Internet Res. 2025;27:e64486. 10.2196/64486. PMC12079073 40305085

[31] Agrawal M, Chen IY, Gulamali F, Joshi S. The evaluation illusion of large language models in medicine. Npj Digit Med. 2025;8:600. 10.1038/s41746-025-01963-x. PMC12504413 41057566

[32] Van Dis EAM, Bollen J, Zuidema W, Van Rooij R, Bockting CL. ChatGPT: five priorities for research. Nature. 2023;614:224–6. 10.1038/d41586-023-00288-7. 36737653

[33] Mannion AF, Kämpfen S, Munzinger U, Kramers-de Quervain I. The role of patient expectations in predicting outcome after total knee arthroplasty. Arthritis Res Ther. 2009;11:R139. 10.1186/ar2811. PMC2787271 19772556

[34] Topol EJ. High-performance medicine: the convergence of human and artificial intelligence. Nat Med. 2019;25:44–56. 10.1038/s41591-018-0300-7. 30617339

[35] Tozsin A, Ucmak H, Soyturk S, Aydin A, Gozen AS, Fahim MA, et al. The Role of Artificial Intelligence in Medical Education: A Systematic Review. Surg Innov. 2024;31:415–23. 10.1177/15533506241248239. 38632898

[36] Picchiarelli A. Artificial Intelligence in Healthcare: Opportunities and Risks. Psychiatr Danub. 2023;35(Suppl 3):90–2. 37994071

[37] Colò G, Leigheb M, Surace MF, Fusini F. The efficacy of shoes modification and orthotics in hallux valgus deformity: a comprehensive review of literature. Musculoskelet Surg. 2024;108:395–402. 10.1007/s12306-024-00839-9. 38922494

