# Supplementary material for: Evaluating the reliability, quality, and readability of AI-generated patient education on hallux valgus: a comparative study of large language models
Source: BMC Med Inform Decis Mak. 2026 Jul 30;26:433. doi: 10.1186/s12911-026-03722-8 (PMC13711935; doi:10.1186/s12911-026-03722-8)
Supplement: Supplementary file 1 [file 12911_2026_3722_MOESM1_ESM.docx]

**HV AI QUESTIONS AND ANSWERS**

# **CHATGPT-4o ANSWERS**

## **1. Etiology and Clinical Presentation**

### **Why does hallux valgus occur?**

Hallux valgus (bunion deformity) is characterized by lateral deviation of the great toe and the formation of a bony prominence (bunion) at its base. Its causes can be grouped into several main categories:

**Genetic Predisposition**

- More common in individuals with a family history.
- Connective tissue laxity, foot bone structure, or joint shape may be inherited.

**Footwear Choice**

- Narrow-toed and high-heeled shoes compress the great toe, predisposing to deformity progression.
- More common in women who wear this type of footwear for prolonged periods.

**Foot Structure and Biomechanical Factors**

- Flatfoot (pes planus) and excessive pronation facilitate hallux valgus development.
- A long first metatarsal bone or structural differences in the joint increase deformity risk.

**Other Factors**

- Inflammatory joint diseases such as rheumatoid arthritis and connective tissue disorders.
- Traumas or post-surgical changes.
- Muscle imbalances.

In summary, the most important cause is genetic predisposition, while improper footwear choice and biomechanical factors accelerate the onset and progression of the deformity.

### **Is hallux valgus genetic? If it runs in the family, can I get it too?**

Yes, genetic factors are very important in the development of hallux valgus.

- The probability of occurrence is significantly higher in individuals with a family history of hallux valgus.
- In particular, foot structure, bone shape, joint flexibility, and connective tissue laxity are hereditary traits that create predisposition to hallux valgus.
- However, not only genetics — environmental factors such as footwear, flatfoot, and rheumatic diseases can also accelerate the process.

In summary, if it runs in your family, the probability of developing it is high, but it is not always certain. Proper footwear choice and attention to foot health can reduce the risk.

### **Is shoe choice (high heels, narrow shoes) effective in the development of hallux valgus?**

Yes, shoe choice is very influential in the development and progression of hallux valgus.

- Narrow-toed shoes: Push the great toe inward, causing finger compression and prominent bony protrusion (bunion).
- High-heeled shoes: Shift body weight forward, increasing the load on the great toe joint and accelerating deformity.
- Rigid-soled shoes: Restrict the natural movement of the foot and increase pressure on the joint.

In summary, shoes alone do not cause hallux valgus (genetics and foot structure also play a role), but they rapidly bring out and advance existing predisposition.

The ideal shoe characteristics to reduce hallux valgus risk are:

- Wide toe box — Toes should be able to move freely without compression.
- Heel height should not exceed 3–4 cm — Excessively high heels load the first metatarsophalangeal joint.
- Made from soft and flexible material — Conforms to foot shape and reduces pressure.
- Supportive sole structure — Especially orthopedic footwear with medial arch support (rear sole support) should be preferred.
- Non-slip and shock-absorbing sole — Distributes pressure evenly and prevents joint loading.
- Should fit properly but not be tight — Overly loose shoes cause friction; overly tight ones cause deformity.
- Lace-up or velcro models — Adjustable to foot volume, providing comfort.

In summary: Wide toe box + low heel + orthopedic sole = the most suitable shoe for hallux valgus.

### **Does flatfoot lead to hallux valgus?**

Yes, flatfoot (pes planus) is an important risk factor in the development of hallux valgus.

- In flatfoot, the medial arch of the foot decreases or disappears entirely.
- This changes the way the foot contacts the ground — pronation (inward rolling) increases.
- Excessive pronation causes abnormal loading on the first metatarsophalangeal joint (great toe base joint).
- Over time, the great toe drifts laterally (toward the little toe), developing hallux valgus.

In summary, individuals with flatfoot — especially in combination with genetic predisposition and improper footwear — are much more likely to develop hallux valgus.

### **Is hallux valgus seen in children?**

Yes, hallux valgus can occur in children too, but it differs from adults:

- Hallux valgus seen in childhood is generally called 'juvenile hallux valgus.'
- It most commonly appears during adolescence (ages 10–15).
- Genetic predisposition is the most important factor in this age group.
- Although footwear choice is less influential in children compared to adults, narrow and pointed-toe shoes can accelerate deformity progression.
- It is more common especially in children with flatfoot.
- Hallux valgus beginning in childhood usually progresses faster and can be more severe in adulthood.

In summary, hallux valgus can occur in children, with risk increasing particularly if there is a family history and if flatfoot coexists.

### **Is pain, swelling, and redness normal in hallux valgus?**

Yes, pain, swelling, and redness are very common findings in hallux valgus and are considered normal.

**Pain:**

- Occurs because the shoe rubs against the bony prominence (bunion) at the great toe base.
- Long walks, narrow/hard shoes, or prolonged standing increase the pain.

**Swelling:**

- Occurs when soft tissue irritation and an inflammatory reaction develop around the joint.
- Swelling may increase especially at the end of the day or after prolonged walking.

**Redness and warmth:**

- Related to skin irritation over the bony prominence.
- Over time, bursitis (inflamed bursa) may develop there.

In summary, pain, swelling, and redness are among the natural manifestations of hallux valgus. However, if pain becomes constant, wakes you from sleep at night, or redness and warmth increase rapidly, a physician should be consulted for additional problems such as infection or advanced bursitis.

## **2. Treatment Options and Surgical Decision-Making**

### **Is non-surgical treatment possible in hallux valgus?**

Yes, non-surgical treatment is possible for hallux valgus; however, these treatments do not completely correct the deformity — they only reduce pain, slow progression, and improve quality of life.

**Non-surgical treatment methods:**

- Footwear choice: Wide-toed, low-heeled, soft-soled shoes should be preferred.
- Orthoses and silicone devices: Interdigital silicone spacers or night splints help maintain a more correct toe position.
- Insole (orthosis) use: Custom insoles especially in patients with flatfoot correct load distribution.
- Physical therapy and exercises: Exercises strengthening the foot muscles (e.g., picking up a towel with the toes, resistance band exercises) may slow deformity progression.
- Medication and cold application: Anti-inflammatory drugs (as recommended by a physician) and ice application may be beneficial for pain and swelling.

**When is surgery needed?**

- If pain limits daily life,
- If wearing shoes becomes difficult,
- If deformity is progressing rapidly, surgery may be considered.

In summary: Complete correction is not expected with non-surgical treatment, but symptoms can be brought under control.

### **Do methods such as orthopedic insoles, splints, pads, exercises, or taping work in hallux valgus?**

In hallux valgus, orthopedic insoles, splints, pads, exercises, and taping provide the following:

**Orthopedic insoles:**

- Correct foot loading and weight distribution.
- Especially in patients with flatfoot or excessive pronation, slow deformity progression.

**Splints and toe pads:**

- Silicone splints used during the day or night maintain the toe in a correct position.
- Reduce friction against the bony prominence and decrease pain.

**Exercises:**

- Strengthen foot muscles and increase toe flexibility.
- Examples: towel picking, marble picking with toes, resistance band exercises.

**Taping:**

- Temporarily positions the toe joint in a correct position.
- Effective only for short-term pain and swelling control; does not provide permanent correction.

**Summary:**

- These methods form the basis of non-surgical treatment.
- They do not completely correct the deformity, but reduce pain, prevent friction, and slow progression.
- If the deformity is advanced and affects daily life, surgery is required.

### **In which cases is surgery necessary for hallux valgus?**

Surgery for hallux valgus is generally considered in the following situations:

**Pain and functional loss:**

- Difficulty wearing shoes, constant pain while walking.
- Failure to achieve sufficient relief with conservative methods (splints, insoles, exercises, analgesics).

**Advanced deformity:**

- If the great toe has shifted significantly toward other toes (generally above a 30–40° angle).
- If the bony prominence (bunion) on the forefoot is prominent and consistently causing problems.

**Joint and toe problems:**

- Toe deformities, hammer toe or overlapping of the second toe.
- If joint stiffness or arthrosis has developed.

**Aesthetic or personal reasons:**

- In cases where the patient experiences significant discomfort even without pain (especially due to social/footwear concerns).

**Recurrent inflammation or bursitis:**

- Conditions of persistent bursa inflammation and swelling and redness next to the great toe.

In summary, pain, functional loss, and advanced deformity are the primary criteria determining the surgical decision. Mild, painless hallux valgus usually does not require surgery and can be controlled with conservative methods.

### **What are the types of hallux valgus surgery?**

Hallux valgus surgeries are performed using different methods depending on disease severity, bone deformity, joint condition, and patient age/activity level. The main surgical types are:

**1. Osteotomy Methods (Bone Cutting and Correction):**

Among the most commonly applied surgical methods for hallux valgus. The deformity is corrected by cutting the metatarsal bone or phalanx (toe bone).

- Distal osteotomy: Applied to the metatarsal bone near the great toe tip. Preferred for mild to moderate hallux valgus.
- Proximal osteotomy: Performed at the base of the metatarsal bone. Used in more severe deformities.
- Chevron osteotomy: The bone is shifted by making a V-shaped cut. Generally applied in mild to moderate hallux valgus.
- Scarf osteotomy: The bone is shifted by making a Z-shaped cut, providing more stable correction. Preferred in moderate to severe deformities.

**2. Arthrodesis (Joint Fusion):**

If the great toe joint is severely damaged or arthritis is present, the joint is stabilized (fused). This method completely eliminates joint pain but results in loss of motion.

**3. Arthroplasty (Joint Prosthesis or Partial Reconstruction):**

- Resection arthroplasty: The deformed joint portion is removed and reshaped with soft tissues. Generally preferred in elderly patients with low activity levels.
- Total joint prosthesis: The great toe joint is replaced with a prosthesis. Provides less pain, better motion, but prosthesis lifespan is limited.

**4. Minimally Invasive / Endoscopic Methods:**

- Methods using small incisions for bone and soft tissue correction.
- Provide less pain, faster healing, and better aesthetic results. However, limited in advanced deformities.

**5. Soft Tissue Interventions:**

- If tendon and ligament imbalances exist, soft tissues around the joint are corrected.
- Usually combined with osteotomy.

Surgeons apply these methods individually or in combination as needed. The type of surgery is determined by the degree of deformity and joint condition.

### **What should I do if my child has the onset of hallux valgus?**

When you notice the onset of hallux valgus in your child, what you can do is very important for slowing deformity progression and preventing pain with early intervention. Here is a detailed guide:

**1. Footwear Choice:**

- Avoid narrow and pointed-toe shoes.
- Prefer wide-toed, flexible shoes that adapt to the natural foot structure.
- Heel height should be minimal (flat-soled shoes if possible).

**2. Orthopedic Supports:**

- Insole (orthopedic inner sole): Supports the bone structure and weight distribution of the foot.
- Night splint: Gently corrects the great toe position during sleep. Generally used short-term and with physician recommendation.

**3. Exercises:**

- Toe opening-closing exercises: Slow deformity progression.
- Ball or towel squeezing exercise: Strengthens foot muscles.
- Interdigital taping: Provides mild correction and support; used temporarily.

**4. Treatment if flatfoot or other foot problems exist:**

- If your child has flatfoot, supporting with orthopedic insoles or physical therapy can reduce hallux valgus progression.

**5. Regular follow-ups:**

- Hallux valgus can progress, so orthopedic check-up every 6–12 months is recommended.
- Keep in mind that deformity can progress rapidly during growth periods.

**6. Consideration of surgery:**

- Surgery is generally not required in children, and surgery is considered after puberty.
- However, if there is severe deformity, pain, or functional loss, surgery may be planned.

In summary: In the early period, slowing hallux valgus progression with proper footwear, supports, and exercises is possible. Surgery is generally considered as a last resort.

## **3. Postoperative Course and Functional Recovery**

### **How long does recovery take after hallux valgus surgery?**

Recovery time after hallux valgus surgery varies depending on the surgical method performed, severity of deformity, and individual factors such as patient age and nutritional status. Generally, it can be summarized as follows:

**1. Immediate Postoperative Period (0–2 weeks):**

- The foot is usually protected with a bandage or cast.
- Swelling and bruising around the great toe are normal.
- Pain is mild to moderate; analgesics prescribed by the physician are used.
- The foot is kept elevated; special post-op shoes or insoles are used when walking if possible.

**2. Early Recovery (2–6 weeks):**

- Bandages or splints are usually removed or changed.
- Light walking is allowed, but running, jumping, or prolonged standing is not recommended.
- Swelling may still continue, especially at the end of the day.

**3. Intermediate Period (6–12 weeks):**

- Most patients can start wearing normal shoes, but narrow shoes and high heels are not recommended.
- Physical therapy or foot exercises may be started.
- Bone healing continues, so full weight bearing may be restricted depending on the surgical method.

**4. Full Recovery (3–6 months):**

- Swelling and pain largely resolve.
- Bone and soft tissues fully heal.
- Patients can usually return to sports and intense activities.
- Rarely, mild swelling or tenderness lasting 6–12 months may occur in some patients.

**Summary:**

- Light daily activities: 2–6 weeks
- Normal shoes and walking: 6–12 weeks
- Full recovery and sports: 3–6 months

### **When can I wear normal shoes after hallux valgus surgery?**

The time to wear normal shoes after hallux valgus surgery varies according to the type of surgery performed and the state of foot healing. The general guide is as follows:

**1. Minimally invasive or mild osteotomy patients:**

- Special post-op or wide-base shoes are generally used for 2–4 weeks.
- Normal shoes: Can be worn 4–6 weeks later with physician approval depending on bone and suture status.

**2. Moderate-degree corrections such as Chevron or Scarf osteotomy:**

- Post-op shoes or cast are used for 4–6 weeks.
- Waiting 6–8 weeks is generally required for normal shoe wear.

**3. Proximal osteotomy or combined procedures:**

- Bandage, splint, or post-op shoe may be required for 6 weeks or longer for foot stability.
- Normal shoes are generally appropriate after 8–12 weeks.

**Extra tips:**

- Shoes should be wide-toed and soft; narrow or hard shoes should be avoided for the first few months.
- Narrow shoes should not be worn as long as swelling continues.
- Following physician and physical therapy recommendations reduces complication risk.

**Summary:**

- Mild correction: 4–6 weeks
- Moderate correction: 6–8 weeks
- Advanced correction or combined surgery: 8–12 weeks

### **Will there be a scar after hallux valgus surgery?**

Yes, scars may remain after hallux valgus surgery, but the technique used and aftercare are important to minimize scarring.

**1. Surgical Site and Scar:**

- The scar is usually on the medial surface of the great toe, in the area where the bony prominence is located.
- With minimally invasive or endoscopic techniques, very small incisions are made, so the scar is less prominent.
- With traditional open osteotomy or combined surgeries, the scar may be slightly longer.

**2. Factors Affecting the Scar:**

- Surgical technique: Minimally invasive methods reduce the risk of scarring.
- Skin type: Scars may be more prominent on darker or oilier skin.
- Age: Scars are less noticeable in younger people as skin is more elastic.
- Healing process and care: Suture care, absence of infection, and sun protection reduce scarring.

**3. Management of Scars:**

- Wound care products, silicone tape, or cream recommended by the doctor may be used.
- Within the first 6–12 months, scars generally soften and fade.
- Aesthetic intervention is rarely needed for very prominent scars.

**Summary:**

- Mild scarring after surgery is normal.
- With modern techniques and proper care, scars are generally minimal and not aesthetically bothersome.

### **How long does it take to return to daily life and work after hallux valgus surgery?**

Return to daily life and work after hallux valgus surgery varies depending on the type of surgery performed, physical requirements of the job, and the patient's recovery speed. It can be summarized as follows:

**1. Desk job or light standing work:**

- Minimally invasive or mild osteotomy patients: Return to daily activities and work may be possible within 1–2 weeks.
- Moderate-degree osteotomy (Chevron, Scarf): Return to work and daily life is generally safe within 2–4 weeks.
- Proximal osteotomy or combined procedures: May take 4–6 weeks.

**2. Physical or intensely standing work:**

- Minimal procedure: 3–4 weeks
- Moderate-degree procedure: 4–6 weeks
- Advanced or combined procedures: 6–8 weeks

**3. Daily life activities:**

- Light walking, housework: Can begin within 1–2 weeks.
- Walking with normal shoes: 6–8 weeks with surgeon approval.
- Sports or intense exercise: 3–6 months depending on surgery performed.

The following table provides an overview:

| **Surgery Type** | **Desk Job** | **Physical Work** | **Sport / Intense Activity** |
| --- | --- | --- | --- |
| Minimally invasive / mild osteotomy | 1–2 weeks | 3–4 weeks | 3 months |
| Chevron / Scarf (moderate) | 2–4 weeks | 4–6 weeks | 3–4 months |
| Proximal / combined | 4–6 weeks | 6–8 weeks | 4–6 months |

### **Does hallux valgus recur after surgery?**

Yes, recurrence after hallux valgus surgery is possible, but it is quite possible to reduce the risk with correct surgical technique and care.

**1. Probability of Recurrence:**

- Various studies have reported hallux valgus recurrence rates between 5–30%.
- The rate varies depending on factors such as severity of deformity, type of surgery, and patient characteristics.

**2. Factors Affecting Recurrence:**

- Preoperative deformity severity: If advanced hallux valgus or metatarsophalangeal angle is very high, recurrence risk increases.
- Bone and joint condition: Arthritic joint or weak bone structure creates risk.
- Surgical technique and surgeon experience: Incorrect osteotomy or insufficient soft tissue balance may cause recurrence.
- Footwear choice and lifestyle: Wearing narrow-toed and high-heeled shoes and continuously loading the foot increases recurrence risk.
- Patients in growth period: If surgery is performed before growth is complete, deformity may redevelop.

**3. Measures to Prevent Recurrence:**

- Selection of appropriate surgical technique (osteotomy and soft tissue correction according to deformity).
- Postoperative care: Bandage, post-op shoes, exercises, and physical therapy.
- Wearing supportive shoes: Wide-toed, low-heeled shoes.
- Regular check-ups: Orthopedic check-up every 6–12 months for early detection of deformity.

**Summary:**

- Hallux valgus can recur after surgery, but risk can be reduced depending on surgical technique, patient compliance, and lifestyle.
- Recurrence risk is lower with minimally invasive methods and appropriate post-op care.

## **4. Long-Term Outcomes and Quality of Life**

### **Does hallux valgus worsen over time if left untreated?**

Yes, hallux valgus can progress and worsen over time if left untreated. However, the speed of progression varies from person to person, and in some individuals it may follow a mild course for years.

**1. Progression of Deformity:**

- The great toe gradually drifts outward and the bony prominence (bunion) on the forefoot grows.
- Pain, redness, and swelling in the toe and foot joints may increase.
- In advanced stages, the toe may lie sideways or override the second toe.

**2. Pain and Functional Loss:**

- Initially, pain may only occur when wearing narrow shoes or during long walks.
- Over time, pain may increase in daily activities and walking.
- Toe movements may become restricted; joint stiffness and arthritis may develop in some patients.

**3. Footwear and Quality of Life:**

- As deformity progresses, wearing narrow shoes becomes difficult.
- Sports and daily activities may be restricted.
- Advanced hallux valgus may also cause aesthetic discomfort.

**4. Risk Factors:**

- Genetic predisposition, flatfoot, wearing narrow and high-heeled shoes, and excess weight accelerate progression.

**Summary:**

- Hallux valgus most often progresses if not treated.
- Early conservative measures (shoe change, insole, exercise) can slow progression, but advanced correction may not be possible without surgery.

### **Does hallux valgus affect other toes?**

Yes, hallux valgus can affect other toes and the overall foot structure, especially as deformity progresses.

**1. Position of Toes and Pressure Distribution:**

- As the great toe drifts laterally, the pressure of the metatarsal bones on the forefoot changes.
- Extra load may form on the second and third toes.
- This may lead to deformities such as hammer toe or mallet toe.

**2. Pain and Hardening on the Forefoot:**

- Calluses and hardened skin may develop on the metatarsal heads (bony prominences at the toe bases).
- Pain and pressure increase on the forefoot become more prominent as hallux valgus progresses.

**3. Shoe Fit:**

- As the great toe drifts laterally, the shoe becomes narrow and other toes are compressed.
- This situation increases the risk of irritation, fungal infections, or calluses between the toes.

**4. Functional Effects:**

- Walking and balance may be disturbed; additional loading on the heel and knee may occur when there is pain on the forefoot.
- Foot muscles and tendons can also be adversely affected in the long term.

**Summary:**

- Hallux valgus affects not only the great toe but also other toes and the general mechanical structure of the foot.
- Early intervention can prevent both great toe deformity and the involvement of other toes.

### **Does hallux valgus affect my walking or playing sports?**

Yes, hallux valgus can affect walking and sports activities, especially as deformity progresses or if pain is present.

**1. Effect on Walking:**

- As the great toe drifts laterally, the forefoot balance is disrupted.
- Pain and discomfort may occur during long walks or on hard surfaces.
- Load may shift to the medial foot or heel, creating additional stress on the knee and hip.

**2. Effect on Sports and Exercise:**

- Sports requiring high foot load such as running, football, basketball may become painful.
- Narrow sports shoes can press on the great toe prominence causing pain and irritation.
- Toe movements may be restricted, impairing balance and performance.

**3. Pain and Swelling:**

- During activity, swelling, redness, and warmth may occur in the hallux valgus region.
- Risks of calluses or fungal infections increase in advanced deformities.

**4. Prevention and Support:**

- Wide-toed and soft-soled shoes should be preferred.
- Orthopedic insoles or night splints can ease activity in some situations.
- If pain exists, muscle support can be provided through physical therapy and exercise.

**Summary:**

- In mild hallux valgus, walking and sports are usually unaffected.
- In moderate to advanced hallux valgus, pain and mechanical changes can limit walking and sports performance.
- Effects can be reduced with early precautions and proper footwear choice.

### **Does losing weight reduce hallux valgus-related complaints?**

Yes, losing weight can reduce hallux valgus-related complaints, but it does not completely correct the deformity.

**1. Reduces Load on the Foot:**

- Excess weight causes more load on the forefoot and great toe.
- When weight is lost, pain and swelling decrease, and walking and daily activities become more comfortable.

**2. Slows Deformity Progression:**

- Hallux valgus progresses with constant pressure applied to the medial foot and great toe.
- As this pressure decreases with weight loss, the progression rate of deformity may slow.

**3. Contribution to Joint and Muscle Health:**

- With less load, foot muscles are less strained, tendons and ligaments relax.
- Load on knee, hip, and lower back joints also decreases, reducing overall pain.

**4. Summary:**

- Weight loss: Reduces pain, eases walking, slows deformity progression.
- Weight loss does not correct the deformity; appropriate footwear, insoles, and surgery if necessary remain important in the presence of hallux valgus.

# **GEMINI-2.5-FLASH ANSWERS**

## **1. Etiology and Clinical Presentation**

### **Why does hallux valgus occur?**

Hallux valgus is a foot deformity characterized by lateral deviation of the great toe and medial protrusion of the bone (metatarsal head) at the base of the toe as a result of this deviation. Multiple factors play a role in its formation.

Here are the most common causes of hallux valgus:

**1. Genetic Predisposition (Most Important Factor)**

The most prominent cause of hallux valgus is genetic predisposition. The majority of patients have a family history. If this problem exists in your family (parents, grandparents), your risk of developing this condition is higher. Foot structure, ligament flexibility, and bone shape such as genetic characteristics can predispose to hallux valgus development.

**2. Footwear Choice**

One of the biggest reasons for its higher frequency in women is footwear choice.

- Narrow and pointed-toe shoes: These shoes compress the foot toes and force the great toe outward, over time causing the formation and progression of the deformity.
- High-heeled shoes: When wearing high-heeled shoes, a large portion of body weight that should normally be on the heel is loaded onto the forefoot and toes. This significantly increased load and pressure on the great toe joint can accelerate deformity development and progression.

**3. Foot Structure Problems**

- Flatfoot (Pes Planus): Flatfoot, which occurs with the collapse of the medial arch of the foot, disrupts the normal biomechanics of the foot and changes the weight distribution in the forefoot. This can increase the pressure on the great toe joint.
- Hypermobility (Joint Laxity): Some people's joints may be more flexible and mobile than normal. This can cause the great toe joint to move abnormally and easily deform.
- Abnormal Foot Biomechanics: Walking or posture disorders can cause unbalanced functioning of foot muscles, creating abnormal stress on the great toe joint.

**4. Rheumatic Diseases**

Inflammatory diseases affecting joints, such as rheumatoid arthritis, can lead to inflammation and deformities in foot joints. This can accelerate hallux valgus development.

**5. Injuries and Traumas**

Blows or traumatic injuries to the great toe can disrupt joint structure and predispose to hallux valgus formation.

In summary, hallux valgus does not have a single cause; it is generally a complex condition that develops on the basis of genetic predisposition triggered by environmental factors such as improper footwear use.

### **Is hallux valgus genetic? If it runs in the family, can I get it too?**

Yes, hallux valgus is thought to be genetic. One of the most important causes of this condition is genetic predisposition.

A family history of hallux valgus (having this problem in close relatives such as parents, grandparents) significantly increases your risk of also developing hallux valgus. This stems not only from the genetic similarity in foot shape, but also from inherited factors such as ligament laxity and the structure of foot joints.

However, genetic predisposition alone is not sufficient for hallux valgus to occur. In people with genetic predisposition, environmental factors such as narrow and pointed-toe shoes can trigger or accelerate the appearance of the deformity. Therefore, people with a family history of hallux valgus are generally recommended to pay particular attention to footwear choice and take care of foot health.

### **Is shoe choice (high heels, narrow shoes) effective in the development of hallux valgus?**

Yes, shoe choice is a highly effective factor in hallux valgus formation. One of the most important reasons it is seen more frequently especially in women is the preference for inappropriate footwear.

Here are details on how shoes contribute to hallux valgus formation:

- Narrow and Pointed-Toe Shoes: These types of shoes force foot toes together away from their natural positions. They push the great toe toward the other toes and create abnormal pressure on the joint. This constant pressure over time causes the great toe to deviate outward and the metatarsal bone to protrude medially (hallux valgus).
- High-Heeled Shoes: When wearing high-heeled shoes, a large portion of body weight, which should normally be on the heel, is loaded onto the forefoot and foot toes. This increased load and pressure significantly increases the stress on the great toe joint. This excessive load on the great toe joint can accelerate the development and progression of the deformity.
- Non-Flexible Soles: Rigid-soled shoes with insufficient flexibility restrict the natural movements of the foot and may disrupt foot mechanics while walking. This can lead to unbalanced functioning of muscles and ligaments in the forefoot, laying the groundwork for deformity formation.

Footwear choice plays a triggering role especially in people with genetic predisposition. For individuals with a family history of hallux valgus, preferring comfortable, wide-toed, flexible-soled, and low-heeled shoes is of great importance in reducing the risk of this deformity.

### **Does flatfoot lead to hallux valgus?**

Yes, flatfoot (pes planus) can lead to hallux valgus or accelerate the progression of an existing condition.

The relationship between these two conditions is closely related to foot biomechanics. Here are explanations on how flatfoot predisposes to hallux valgus:

- Change in Foot Structure: Flatfoot is the condition of the medial arch of the foot collapsing and the sole of the foot making full contact with the ground. This collapse causes the ankle to rotate inward (pronation). This abnormal movement changes the alignment of bones and joints in the forefoot.
- Abnormal Weight Distribution: As a result of foot pronation, body weight falls more on the medial side of the foot, especially on the area where the great toe and first metatarsal bone are located, compared to normal. This abnormal weight distribution increases the stress on the great toe joint.
- Muscle and Ligament Imbalance: Flatfoot causes the muscles and tendons on the medial side of the foot to be overstretched and the muscles on the lateral side to weaken. This muscle imbalance pulls the great toe medially, facilitating deformity formation.

Therefore, although flatfoot alone may not cause hallux valgus, it can trigger the onset of the disease and accelerate its progression when combined with other factors such as genetic predisposition. For people with flatfoot to reduce hallux valgus risk, taking measures such as orthopedic insoles (orthoses) or appropriate footwear choice is generally recommended.

### **Is hallux valgus seen in children?**

Yes, hallux valgus can also be seen in children, but it appears less frequently and for different reasons compared to adults.

The main characteristics and causes of hallux valgus in children are as follows:

- Congenital Conditions: In some children, hallux valgus may be congenital. This condition results from genetic predisposition or anomalies in the developmental process. These cases are usually noticed at an early age and can sometimes be treated with non-surgical methods (splinting, exercise, etc.).
- Flatfoot and Gait Disorders: One of the most common causes in children is flatfoot (pes planus). Flatfoot disrupts the normal structure of the foot, placing abnormal load on the great toe, and can lead to hallux valgus over time. In addition, other gait disorders such as toeing in or toeing out can also trigger this condition.
- Footwear Choice: Although not as prominent a cause as in adults, children wearing narrow and tight shoes inappropriate for foot development can contribute to the formation of deformity.

**Treatment Differences in Children**

Treatment of hallux valgus in children differs from adults because the child's skeletal system is still developing.

- Priority for Conservative Treatment: In children, non-surgical treatment methods (conservative treatment) are first preferred. These methods include foot exercises, custom-made orthopedic insoles, and night splints. These treatments aim to stop or slow deformity progression.
- Surgical Decision: Surgery is generally considered only in very severe cases or when conservative treatments have not worked. For surgery, the child's skeletal development is expected to be largely complete; otherwise the deformity may recur. The surgical decision should be carefully made by an experienced orthopedist based on the status of the growth plates.

In summary, hallux valgus is seen in children but, unlike in adults, is generally associated with congenital structural problems or developmental causes such as flatfoot rather than environmental factors like footwear use. In treatment, priority is also given to non-surgical methods.

### **Is pain, swelling, and redness normal in hallux valgus?**

Yes, pain, swelling, and redness are quite common and considered normal symptoms in hallux valgus. These symptoms arise from the pressure and irritation caused by the deformity in the great toe.

The causes of these symptoms are as follows:

- Pain: The deformed great toe joint (metatarsophalangeal joint) is constantly exposed to friction and pressure during activities such as walking, standing, or wearing shoes. This causes chronic inflammation and pain in the joint. The severity of pain can vary depending on the degree of deformity and the type of footwear worn.
- Swelling and Redness: The bone protruding in the shoe constantly rubs against the inner surface of the shoe. This rubbing and pressure causes irritation of the skin and inflammation of the tissues around the joint. This inflammation in turn causes swelling and redness (bursitis) around the joint. This condition where pain, swelling, and redness are present together is generally called a 'bunion'.
- Callus and Hardening: As a result of constant rubbing and pressure, the skin thickens to protect itself and calluses or hardened skin tissue forms. This condition also makes walking and wearing shoes difficult.

These symptoms are important signs that hallux valgus is progressing and needs to be treated. In case of severe pain and inflammation, it is important to consult a physician to stop the progression of the deformity and improve the patient's quality of life.

## **2. Treatment Options and Surgical Decision-Making (Gemini)**

### **Is non-surgical treatment possible in hallux valgus? (Gemini)**

Yes, non-surgical treatment of hallux valgus is possible, but depends on the degree of the condition and the patient's symptoms. Non-surgical treatment methods are generally preferred in the early stages of the disease, when pain is mild, and in cases where deformity has not progressed much.

The primary goal of non-surgical treatment is to reduce pain, slow deformity progression, and improve the patient's quality of life.

The most commonly used non-surgical treatment methods are:

- Appropriate Footwear Choice: This is the most basic and most important step. Wide-toed, comfortable, soft-leather, and low-heeled shoes should be preferred. This reduces pressure on the great toe and alleviates pain. High-heeled or pointed-toe shoes should be avoided.
- Orthopedic Insoles (Orthotics): Custom-prepared insoles regulate the biomechanical balance of the foot, optimizing weight distribution. This reduces abnormal load on the great toe joint and helps relieve pain.
- Interdigital Spacers or Silicone Separators: Silicone separators placed between the great toe and the second toe prevent the toes from rubbing against each other and help maintain the great toe in a more correct position. They also reduce friction in the shoe, preventing callus and hardening formation.
- Night Splints (Bunion Splints): These splints used during sleep apply mild pressure to keep the great toe in the correct position. The aim of these splints is to slow deformity progression and reduce morning stiffness. However, it should be known that these splints do not permanently correct the deformity.
- Medications and Cold Compresses: In case of pain and inflammation, anti-inflammatory drugs (analgesics) may be used with the physician's recommendation. Also, cold compresses applied to the painful area are effective in reducing swelling and pain.
- Physical Therapy and Exercises: Special exercises aimed at strengthening foot muscles and increasing flexibility can slow deformity progression and relieve pain.

It should be remembered that non-surgical treatment methods cannot completely correct the bone deformity caused by hallux valgus. These treatments are generally used to control symptoms and delay the need for surgery. In situations where deformity has progressed and seriously affects daily life (severe pain, difficulty walking, etc.), surgical treatment may be required for a permanent solution.

### **Do methods such as orthopedic insoles, splints, pads, exercises, or taping work in hallux valgus? (Gemini)**

Yes, orthopedic insoles, splints, pads, exercises, and taping methods work in hallux valgus treatment. However, the purpose of these methods is to relieve symptoms, reduce pain, and slow disease progression rather than completely correcting the deformity.

The functions of each of these methods are:

- Orthopedic Insoles (Orthotics): Corrects biomechanical problems of the foot such as flatfoot, balancing weight distribution on the foot. This reduces abnormal pressure on the great toe joint especially while walking, relieving pain and potentially slowing deformity progression. Custom-made insoles provide the most effective results.
- Splints and Taping: Night splints are generally used during sleep and ensure the joint rests by keeping the great toe in the correct position. May slow deformity progression and reduce morning pain. Taping: Special taping techniques applied by physiotherapists can help reduce pain and temporarily correct joint position by supporting the great toe. However, this is not a permanent solution.
- Pads (Bunion Protectors): Pads usually produced from silicone material reduce friction inside the shoe. They prevent pain and redness resulting from friction of the protrusion known as bunion against the shoe. In addition, pads placed between the toes also prevent the toes from rubbing against each other, providing relief.
- Exercises: Exercises aimed at strengthening foot and toe muscles, especially working the plantar foot muscles, help support the foot arch. These exercises can reduce stress on the great toe joint by correcting muscle imbalance and can slow deformity progression. For example, pulling a towel or rolling a small ball with foot toes are frequently recommended exercises.

These methods provide important support especially in the early stages of the disease and for patients wishing to avoid surgery. However, if the deformity is advanced and seriously affects daily life, surgical treatment should be considered for a permanent solution. Therefore, consulting an orthopedic specialist to decide which method is appropriate for you is the most correct approach.

### **In which cases is surgery necessary for hallux valgus? (Gemini)**

In hallux valgus treatment, surgery is generally considered when conservative (non-surgical) treatment methods are insufficient and the disease begins to negatively affect daily life. Several important factors are considered when making the surgical decision.

The main situations where surgery is necessary for hallux valgus are:

- Severe and Persistent Pain: The most important reason for surgery is the patient experiencing severe pain despite using appropriate footwear and non-surgical treatment methods (insoles, splints, etc.), and this pain seriously restricting daily activities (walking, standing).
- Deformity Progression: Surgery is recommended when the deformity becomes visibly worse over time and reaches an advanced level such as the great toe sliding under or over other toes. This condition can also lead to deformities in other toes (hammer toe, claw toe).
- Difficulty Walking and Activity Restriction: The deformity disrupting the walking pattern and preventing physical activities such as sports is an important reason for surgery. The patient's decreased quality of life may make surgical intervention inevitable.
- Limited Footwear Choice: The situation where the patient can barely wear any shoes comfortably due to the protrusion on the foot and this affecting social life is a condition requiring surgery.
- Stiffness and Movement Restriction in the Joint: Arthrosis (calcification) may develop in the great toe joint as hallux valgus progresses. This restricts joint mobility and cannot be corrected with non-surgical methods.

Important Note: Hallux valgus surgery is generally not performed for cosmetic reasons. The main goal is to eliminate pain, correct foot function, and improve the patient's quality of life. The surgical decision is made jointly by the patient and physician as a result of a detailed evaluation including the patient's symptoms, physical examination findings, and X-ray films.

### **What are the types of hallux valgus surgery? (Gemini)**

In hallux valgus surgeries, many different techniques are used depending on the degree of deformity, patient age, bone structure, and surgeon preference. Generally, surgeries focus on bringing the great toe into correct position and removing the bony prominence.

Hallux valgus surgeries can generally be divided into three main categories:

**1. Distal Osteotomies (Cuts at the End)**

These surgeries are generally preferred in mild to moderate deformities. A small cut is made close to the end of the metatarsal bone (the long bone going to the great toe).

- Chevron Osteotomy: The head of the bone is moved laterally with a V-shaped cut and fixed in the correct position. This is one of the most commonly performed surgeries.
- Mitchell Osteotomy: Involves correction of the bone with a different cut shape and fixation with screws.

**2. Shaft Osteotomies (Cuts at the Middle)**

These techniques are used in more advanced and severe cases of hallux valgus.

- Scarf Osteotomy: The entire metatarsal bone is moved with a Z-shaped cut. This allows both the angle and length of the bone to be corrected.
- Lapiplasty (1st Tarsometatarsal Joint Fusion): Used especially in conditions seen together with flatfoot or in feet that widen excessively. The joint at the base of the metatarsal bone is frozen (fusion). This method significantly reduces recurrence risk by eliminating the main cause of deformity.

**3. Arthrodesis (Joint Freezing) and Arthroplasty (Joint Replacement)**

- Arthrodesis (Fusion): The procedure of freezing the great toe joint. Preferred in patients with severe deformity and calcification (arthrosis) in the joint. This method permanently eliminates pain but completely limits joint mobility.
- Arthroplasty (Joint Replacement): The procedure of removing part or all of the joint and replacing it with a prosthesis in elderly patients or those with severe joint damage. This is a method no longer frequently preferred.

**4. Additional Information**

- Minimally Invasive (Closed) Surgeries: In these techniques developed in recent years, the bone is corrected with special instruments by making small incisions in the skin. The recovery process may be faster but is not appropriate for every case and requires the surgeon to be specialized in this area.
- Fixation of Graft: Bone pieces cut and corrected during surgery are fixed in their new positions using screws, wires, or plates. These implants generally stay inside but can sometimes be removed later depending on the patient's complaint.

The decision on the type of surgery depends on the surgeon's experience, the patient's X-ray findings, and foot structure. The most appropriate method is determined by the physician according to the patient's condition.

### **What should I do if my child has the onset of hallux valgus? (Gemini)**

If you think your child has the onset of hallux valgus or you have noticed a deformity, it is important to plan your steps without panicking.

Here are the steps to follow in this situation:

**1. Consult an Orthopedic Specialist**

The first and most important step is to see a physician specialized in pediatric orthopedics or foot and ankle surgery. The physician will physically examine your child's foot and may request X-rays. This provides important information such as the degree of deformity, bone structure, and status of growth plates. This evaluation is of critical importance for creating the correct treatment plan.

**2. Pay Attention to Appropriate Footwear Choice**

Until and after consulting your physician, the most important preventive step is to pay attention to the shoes your child wears.

- Wide-Toed Shoes: Shoes should be preferred where the toes are not compressed and can move comfortably.
- Flexible and Supportive Sole: The sole of the shoe should be flexible but at the same time should support the foot arch.
- Heelless Shoes: High-heeled shoes should definitely be avoided for children.

**3. Consider Conservative Treatments**

With the physician's recommendation, non-surgical treatment methods can be started. These methods aim to slow deformity progression and keep pain under control.

- Orthopedic Insoles: If there are accompanying problems such as flatfoot, custom insoles may be recommended.
- Toe Separators or Splints: In appropriate situations, silicone separators or splints can be used to separate the toes or maintain correct position while sleeping at night.
- Exercises: Simple exercises aimed at strengthening foot muscles can help support foot structure. A physician or physiotherapist will guide you in this regard.

**4. Observation and Regular Follow-up**

In children, hallux valgus should be regularly monitored as skeletal development continues. Your physician will plan regular check-up appointments at certain intervals to monitor the course of deformity. These follow-ups are important for deciding whether to make changes to the treatment plan.

**5. Delaying Surgery**

In children, surgery is generally considered as a last resort. The biggest reason for this is that growth plates are open and surgery may damage these plates, potentially causing growth problems. Therefore, surgical intervention is generally delayed until the child's skeletal development is complete and is evaluated at an early age only in situations such as very severe pain or difficulty walking.

In summary, if you notice the onset of hallux valgus in your child's foot, you should immediately consult a specialist to have the situation evaluated, and focus on conservative treatment methods with their guidance.

## **3. Postoperative Course and Functional Recovery (Gemini)**

### **How long does recovery take after hallux valgus surgery? (Gemini)**

Recovery time after hallux valgus surgery varies depending on the surgical technique applied, severity of the deformity, the patient's general health condition, and how well postoperative instructions are followed.

On average, the recovery process can be summarized as follows:

**First 1–2 Weeks: Early Recovery Period**

- Pain and Swelling: Pain may occur in the first few days after surgery. This pain is controlled with analgesics prescribed by the physician. Swelling of the foot is normal. Keeping the foot elevated and applying ice are important for reducing swelling.
- Walking: Most patients are given a specially designed rigid-soled surgical shoe (post-op shoe). With this shoe, controlled weight-bearing and walking can generally begin immediately after surgery or within a few days. This shoe is generally used for 2–6 weeks.
- Dressing: The surgical wound dressing is changed regularly. Sutures are usually removed 1–2 weeks later.

**2–6 Weeks: Intermediate Recovery Period**

- Surgical Shoe: The patient continues to use the special surgical shoe during this period.
- Daily Activities: Gradual return to daily activities. Prolonged standing and long walks should be avoided.
- Physical Therapy: In some cases, physical therapy may be started to regain toe mobility and strengthen muscles.

**6 Weeks – 3 Months: Advanced Recovery Period**

- Transition to Normal Shoes: During this period, comfortable, wide shoes can generally be started with physician approval. High-heeled or narrow shoes should be avoided.
- Activity Increase: Daily activity level is gradually increased. Return to light sports (swimming, cycling) is possible.
- Swelling: Mild swelling may continue for 3–6 months, and in some cases up to a year.

**3 Months – 1 Year: Full Recovery**

- Full Function: During this period, the foot fully heals and the patient can fully return to normal activities, including sports.
- Final Shape: The foot takes its final shape and surgical scars, if any, lose their prominence.

In summary, the first few weeks after surgery are the most critical period. While most patients can largely return to daily life within 6 weeks, full healing and final shaping of the foot can range from 3 months to 1 year. Fully complying with the physician's instructions is of vital importance for a successful recovery process.

### **When can I wear normal shoes after hallux valgus surgery? (Gemini)**

Starting to wear normal shoes after hallux valgus surgery is an important stage of the recovery process and this time can vary from person to person. Two main factors influence this decision:

- Surgical Technique: The type of surgery performed and how well the bone is stabilized.
- Recovery Rate: The patient's recovery process and the state of swelling.

In general, the process of wearing normal shoes after surgery includes the following steps:

- First 1–6 Weeks: Surgical Shoe (Post-op shoe): Immediately after surgery, a specially designed surgical shoe (post-op shoe) is given that protects the great toe and reduces the load on the forefoot. This shoe allows the patient to walk while protecting the surgical site. Most patients use this shoe for the first 4 to 6 weeks.
- After 6–8 Weeks: Transition to Wide and Comfortable Shoes: With your physician's approval, transitioning from the special surgical shoe to normal shoes is possible. Shoes preferred at this stage should be: Wide-toed (foot structure that allows toes to move freely without compression), Soft and Flexible Materials (upper part of the shoe (leather or fabric) should be flexible and should not press on the surgical site), Low-heeled or Flat-soled (high heels should definitely be avoided as they will increase the load on the forefoot).
- After 3–6 Months: Trying Various Types of Shoes: During this period, swelling largely decreases and the patient can start trying different types including sports shoes. However, comfort should still be the priority.
- After 6 Months – 1 Year: Full Return: When the foot fully heals, it becomes possible for the patient to return to old shoes (if they are appropriate). However, wearing high-heeled and narrow shoes for extended periods is still not recommended as it may cause recurrence of the deformity.

Important Note: Swelling is one of the symptoms that can continue for the longest time after surgery. It can make wearing shoes difficult. Therefore, it is important to wait for the swelling in your foot to substantially decrease before starting to wear normal shoes. To determine the most accurate timing, you must definitely follow the recommendations of the surgeon who performed your surgery.

### **Will there be a scar after hallux valgus surgery? (Gemini)**

Yes, scars remain after hallux valgus surgery. However, with modern surgical techniques, these scars are usually minimal and lose their prominence over time.

The condition of the scar varies depending on several factors:

- Surgical Technique: In open surgical methods (surgeries with wide incisions), the scar may be longer and more prominent. In minimally invasive (closed) surgeries, incisions are very small so scars may be nearly invisible.
- Patient's Skin Type: Everyone's skin heals from wounds differently. While scars heal more easily and quickly in some people, some may be more prone to keloid (raised scar) formation.
- Wound Care: Wound care in the postoperative period is very important. Keeping the wound site clean in accordance with the physician's instructions reduces infection risk and provides better healing. After sutures are removed, the healing process can be supported with scar cream or massage.
- Sun Protection: When the scar is first formed, exposure to sunlight can cause its color to darken. Therefore, protecting the scar from the sun in the first months after surgery is important.

Generally, the surgical scar is located on the medial side of the foot, at the level of the great toe joint. For most patients, this scar fades over time and becomes less visible. To minimize the appearance of the surgical scar, following all care instructions recommended by your physician is the most correct approach.

### **How long does it take to return to daily life and work after hallux valgus surgery? (Gemini)**

Return to daily life and work after hallux valgus surgery shows large differences depending on the type of work and surgical technique. On average, this period can be evaluated as follows:

**For Jobs Requiring Standing and Walking (factory worker, teacher, waiter, etc.)**

Return to work time may be longer for patients working in these professions.

- Average Return Time: 6 to 12 weeks
- Reason: Standing or walking for long periods may cause excessive pressure on the surgical site, leading to increased pain and swelling. Returning to work before the foot fully heals may jeopardize the success of the surgery.

**For Desk or Sitting Jobs (office worker, software developer, etc.)**

Patients working in these types of jobs can generally return to work faster.

- Average Return Time: 2 to 4 weeks
- Reason: As there is no load on the foot, the recovery process proceeds more comfortably. Patients can move with the special surgical shoe within 1–2 weeks after surgery and can go to work with this shoe.

**Other Factors Affecting the Return Process:**

- Surgical Technique: Recovery may be faster with minimally invasive (closed) methods. Surgeries with bone cutting (osteotomy) and fixation with screws may require a longer recovery process.
- Individual Recovery Rate: Everyone's body heals at a different speed. Recovery process may be prolonged in patients with additional health problems such as diabetes or circulation problems.
- Compliance with Physician's Instructions: Resting the foot after surgery, keeping it elevated, and regularly performing prescribed exercises directly affects the recovery process.

Important Note: Return to work is not only related to pain resolution. Reduction of swelling in the foot, complete wound closure, and ability to walk safely are also important. Therefore, you should not make the return to work decision alone; you must definitely talk with the physician who performed your surgery and get their approval.

### **Does hallux valgus recur after surgery? (Gemini)**

There is a risk of disease recurrence (relapse) after hallux valgus surgery, but this risk depends on many factors.

The main factors affecting relapse risk are:

**Surgical Technique: The most impactful factor on relapse risk is the surgical method applied.**

- Incorrect Technique: Selection of a surgical method not appropriate for the patient's degree of deformity increases the risk of disease recurrence. For example, if a technique used in mild cases is preferred for advanced deformity, the probability of relapse increases.
- Anatomical Correction: Surgeries that correct the root cause of the deformity (instability in the first metatarsal bone) such as Lapiplasty have the potential to minimize relapse risk.

**Postoperative Care:**

- Footwear Choice: Re-using narrow, pointed-toe, and high-heeled shoes after surgery again increases pressure on the great toe joint, laying the groundwork for deformity recurrence.
- Physical Therapy: Neglecting exercises and physical therapy program recommended by the physician can lead to weakening of foot muscles and disruption of biomechanical balance.

**Patient's Structural Characteristics:**

- Genetic Predisposition: In people with a family history of hallux valgus, disease recurrence risk may be higher.
- Joint Laxity: In people with excessive joint flexibility (hypermobility), it is possible for the surgically corrected structure to gradually relax over time and the deformity to return.
- Age: Relapse risk may be slightly higher in younger patients due to the bone remodeling potential.
- Disease Severity: The more severe the preoperative deformity, the higher the postoperative relapse risk.

In summary, the success of hallux valgus surgery depends not only on the surgeon's skill but also on the selection of the correct technique and the patient's meticulous adherence to postoperative instructions. Consistently wearing comfortable and appropriate shoes after surgery is the most important step to minimize relapse risk. Going to regular physician check-ups can also enable early detection of a possible relapse.

## **4. Long-Term Outcomes and Quality of Life (Gemini)**

### **Does hallux valgus worsen over time if left untreated? (Gemini)**

Yes, hallux valgus generally worsens and progresses over time if left untreated. Disease progression can vary from person to person depending on the degree of deformity, the patient's lifestyle (such as shoe choice), and genetic predisposition.

Conditions that may occur when left untreated include:

- Increasing Deformity: Lateral deviation of the great toe gradually increases. This can cause the great toe to slide under or over the second toe.
- Worsening Pain and Swelling: Initially mild pain and swelling become more severe and permanent as deformity progresses. This can seriously restrict daily activities (walking, standing).
- Deformities in Other Toes: The push of the great toe can cause the second toe to lose its normal position and rise upward, leading to deformities such as 'hammer toe' or 'claw toe.' This can also cause pain and callus formation in other toes.
- Gait Disorders: As deformity progresses, the patient's walking pattern changes. Weight-bearing on the great toe becomes difficult and the patient tries to transfer weight to the outer side of the foot. This can cause knee, hip, and lower back pain.
- Permanent Joint Damage (Arthrosis): Abnormal pressure on the great toe joint can cause wear of joint cartilage and calcification (arthrosis). This restricts joint mobility and leads to severe pain. Developed arthrosis may not be fully correctable even with surgery and may require more extensive surgeries such as joint fusion.

Important Note: Conservative measures such as appropriate footwear choice, insole use, and exercises in the early stage are of great importance for slowing hallux valgus progression. When symptoms such as pain and difficulty walking appear, consulting a specialist to evaluate treatment options is a critical step to prevent further problems.

### **Does hallux valgus affect other toes? (Gemini)**

Yes, as hallux valgus progresses it can adversely affect other toes and lead to deformities in them.

The main reasons for this interaction are:

- Pressure on the Second Toe: Hallux valgus causes lateral deviation of the great toe. This deviation puts pressure on or under the second toe. Initially just a pressure sensation, over time it causes the second toe's shape to deform and its joints to bend.
- Hammer Toe or Claw Toe Deformity: As a result of the great toe pushing, the second, third, or fourth toes may rise upward and turn into deformities such as 'hammer toe' (bending of toes at the middle joint) or 'claw toe' (toes curling at the tip to look like a claw). These conditions can cause pain, callus, and ulcer formation due to friction while wearing shoes.
- Change in Weight Distribution: As hallux valgus progresses, patients begin to transfer their weight toward the outer side of the foot rather than bearing weight on the great toe. This abnormal weight distribution increases pressure on other toes and the rest of the foot. This leads to problems such as pain in other toes, callus formation, and metatarsalgia (forefoot pain).
- General Disruption of Foot Structure: Hallux valgus causes widening of the forefoot and general disruption of foot structure. This causes other toes to lose their anatomically correct alignment and come into contact with each other.

For this reason, hallux valgus should not be seen as a problem limited to the great toe. If left untreated, it can completely disrupt foot anatomy, creating a domino effect and leading to permanent deformities in other toes. Early intervention is of critical importance to prevent these problems from occurring.

### **Does hallux valgus affect my walking or playing sports? (Gemini)**

Yes, hallux valgus can seriously affect both your walking and your ability to do sports. As deformity progresses, these effects become more prominent and can significantly reduce daily quality of life.

**Effects on Walking**

- Pain and Discomfort: Pain and swelling caused by hallux valgus are felt with every step. It makes wearing shoes difficult and can make prolonged standing or walking impossible.
- Abnormal Walking Pattern: To avoid pain, patients change their natural walking patterns. The support normally taken on the great toe for push-off force decreases due to hallux valgus. This leads to pressing toward the outer side of the foot or inability to get sufficient push force from the toes.
- Other Joint Problems: These changes in walking pattern can cause abnormal load on joints in other areas of the body such as the knee, hip, and lower back. Over time, this can lead to secondary problems such as pain and arthrosis (calcification) in these areas.

**Effects on Playing Sports**

- Performance Loss: Sports activities such as running, jumping, or rapid direction changes require a high level of push force from the forefoot and toes. As hallux valgus reduces this push force, it causes a decrease in sports performance.
- Pain and Injury Risk: High stress placed on the foot during sports intensifies the pain caused by hallux valgus. In addition, disruption of foot biomechanics increases stress on other joints, raising the risk of new injuries (for example; ankle sprains, tendon inflammations).
- Difficulty Finding Appropriate Footwear: Even sports shoes may not be comfortable for a person with hallux valgus. This makes doing sports difficult or completely prevents it.

Important Note: Hallux valgus is not just a cosmetic problem. Left untreated, it causes serious limitations in both daily life and sports activities. If pain and deformity are preventing you from doing sports, consulting an orthopedic specialist to evaluate surgical options can be an important step to regain quality of life.

### **Does losing weight reduce hallux valgus-related complaints? (Gemini)**

Yes, losing weight can reduce hallux valgus-related complaints. Since body weight directly affects the load on the feet and especially the forefoot, weight control plays an important role in relieving symptoms.

Here are the positive effects of losing weight on hallux valgus:

- Reduction of Load on the Feet: Excess weight increases the pressure on the feet with every step. This causes more stress to be applied on the great toe joint and surrounding tissues where the hallux valgus deformity is located. With weight loss, this load decreases, which directly relieves symptoms such as pain and inflammation.
- Improvement of Walking Biomechanics: Foot pronation (inward collapse of the foot) is more common in overweight people. This condition supports the development and progression of hallux valgus. Losing weight can help the foot structure function more balanced and can reduce pressure on the great toe joint by improving walking biomechanics.
- Increase in Mobility: Losing weight increases general mobility and flexibility. This not only reduces foot pain but also makes it easier to do sports and exercise. Regular exercise can provide additional benefits in slowing hallux valgus progression by strengthening foot muscles.
- Postoperative Recovery: For patients considering surgical treatment, losing weight before surgery can increase the success of surgery and accelerate postoperative recovery.

Important Note: Losing weight alone does not correct the bone deformity caused by hallux valgus. However, it is very effective in reducing complaints such as especially pain, swelling, and inflammation, and increases the success of conservative treatment methods. Therefore, reaching and maintaining a healthy weight is an important part of the treatment plan for patients with hallux valgus.

# **DEEPSEEK-V3 ANSWERS**

## **1. Etiology and Clinical Presentation**

### **Why does hallux valgus occur?**

Hallux valgus (bunion deformity) occurs through a combination of genetic, anatomical, and environmental factors. Here are the most common causes:

**1. Genetic Predisposition**

- Family history (genetic transmission exists in 60–80% of cases).
- Congenital foot structure (e.g., flatfoot, hyperflexible joints).

**2. Incorrect Footwear Choice**

- Narrow-toed shoes: Compress the great toe inward.
- High-heeled shoes: Shift weight forward, pressing on the toes.

**3. Anatomical Factors**

- Metatarsus primus varus: Outward deviation of the 1st metatarsal bone.
- Hypermobility: Joints being more flexible than normal.
- Flatfoot (pes planus): Collapse of the foot arch loads the toes.

**4. Neuromuscular Diseases**

- Conditions leading to muscle imbalance such as cerebral palsy, stroke.
- Rheumatoid arthritis (causes joint deformation).

**5. Age and Gender**

- 10 times more common in women (due to hormones and footwear habits).
- Ligament laxity and degenerative changes with aging.

**6. Trauma or Overuse**

- Ankle sprains, repetitive strains (ballet dancers, athletes).

**Summary:**

Hallux valgus usually develops through a combination of genetic and environmental factors (especially footwear). For prevention, orthopedic footwear and exercises from an early age are recommended if genetic predisposition exists.

### **Is hallux valgus genetic? If it runs in the family, can I get it too?**

**Short Answer:**

Yes, hallux valgus is largely associated with genetic predisposition. If it exists in your family (especially in parents or siblings), the risk of it occurring in you increases by 60–80%. However, genetics alone is not solely responsible; environmental factors also play a triggering role.

**Detailed Explanation:**

**1. How Effective Is Genetic Predisposition?**

- Research shows that the frequency of the same deformity in first-degree relatives of patients with hallux valgus is very high.
- Transmission from the mother is particularly more prominent (since it is more common in women).
- Congenital foot structure (such as flatfoot, metatarsus primus varus) can be transmitted genetically.

**2. 'It Runs in My Family, Will I Definitely Get It?'**

- No, it's not certain! Even with genetic predisposition, environmental factors (footwear choice, weight, activity level) determine whether the disease will occur.
- Example: If someone with a family history of hallux valgus doesn't wear narrow shoes, the protrusion may not form.

**3. Which Genes Are Involved?**

- Mutations in collagen genes such as COL1A1 and COL2A1 are thought to cause connective tissue laxity.
- Genes related to skeletal development such as FLNB are also being researched.

**What Can You Do to Reduce Your Risk?**

- Footwear Choice: Avoid narrow-toed and high-heeled shoes. Use orthopedic insoles.
- Exercises: Do exercises that strengthen great toe muscles such as towel curl, toe spread.
- Early Precaution: If it runs in the family, have your foot structure checked from adolescence.

**Conclusion:**

- If you have genetic risk, taking precautions can delay or prevent deformity.
- If complaints begin, consult an orthopedic specialist early (non-surgical treatments are possible).

### **Is shoe choice (high heels, narrow shoes) effective in the development of hallux valgus?**

Definitely YES! After genetic predisposition, footwear choice is the second most important risk factor in hallux valgus formation. Especially narrow-toed and high-heeled shoes place abnormal load on the great toe, triggering the deformity. Here are some striking facts:

**1. What Do Scientific Data Say?**

- The main reason for its 10 times higher frequency in women is footwear preference (Journal of Foot & Ankle Research).
- The risk of hallux valgus increases by 90% in those who wear shoes with heels higher than 5 cm (American Journal of Epidemiology).
- In those wearing narrow shoes, the hallux valgus angle has been measured on average 8–10 degrees more.

**2. How Does It Deform? The Mechanism:**

- High heel: Shifts weight forward, placing excessive load on the metatarsal heads. Example: With a 7 cm heel, the load on the forefoot rises to 76% (normally 43%).
- Narrow toe box: Strains the ligaments by compressing the great toe outward. Over time the great toe is pushed outward and the bone inward → protrusion forms.

**3. Which Shoes Are Most Risky?**

| **Shoe Type** | **Risk Level** | **Examples** |
| --- | --- | --- |
| Stiletto heel (Narrow toe + >7 cm) | ⚠️⚠️⚠️ Very high | Classic high-heeled pumps |
| Ballet flats (Narrow-toed flat shoe) | ⚠️⚠️ Moderate-high | Ballet shoe style |
| Sports shoe (Wide-toed) | ✅ Low | Running shoes, orthopedic sneaker |

**4. Practical Guide with Critical Questions:**

- Q: 'Are flat shoes with narrow toe also risky?' → Yes! Narrow toe box alone is a risk factor (the reason for its frequency in ballet dancers).
- Q: 'How many cm of heel is safe?' → Under 3 cm is ideal. 5 cm+ is the risk threshold.
- Q: 'Is footwear effective in men too?' → Yes! It is also seen in men wearing tight leather shoes (but 1/10 of women).

**5. Four Golden Rules When Choosing Shoes:**

- Toe width: Make sure your great toe can move comfortably.
- Heel height: Don't exceed 3 cm for daily use.
- Sole support: Use orthopedic insoles with arch support.
- Material: Prefer flexible, breathable materials (avoid hard leather).

**Last Word:**

'It's not your genetic destiny!' Even if it runs in your family, you can reduce the risk by up to 50% with proper footwear and exercise. If you have mild complaints right now, you can stop progression with a silicone interdigital spacer and night splint.

### **Does flatfoot lead to hallux valgus?**

Yes, flatfoot significantly increases the risk of hallux valgus. However, this does not mean 'all people with flatfoot develop hallux valgus.' The mechanical relationship between the two and prevention measures:

**1. How Does It Trigger? Anatomical Relationship**

- When the foot arch collapses (flatfoot), the tibialis posterior tendon weakens → excessive pronation (inward rolling) occurs.
- This situation: Causes overloading in the 1st metatarsal bone → metatarsus primus varus (inward rotation) develops. The great toe is pushed toward other toes → hallux valgus angle increases.

Scientific Data: Flatfoot is a concomitant finding in 40–58% of patients with hallux valgus (Journal of Foot and Ankle Surgery).

**2. Who Is at Risk?**

- Those with flexible flatfoot (from childhood).
- Adults with acquired flatfoot (tibialis posterior tendon insufficiency).
- Overweight individuals (places extra load on the foot arch).

**3. What Should Be Done to Prevent It?**

- A) Orthopedic Interventions: Supported insole: Special designs raising the medial arch (carbon fiber or thermoplastic). UCBL-type orthosis: Used in severe flatfoot.
- B) Exercises: Towel curls: Pulling a towel placed under the foot with the toes. Arch lifts: Contracting the foot arch and holding for 5 seconds.
- C) Footwear Choice: Motion control shoes: Hard-soled models limiting pronation (e.g.: Brooks Beast, Asics Gel-Kayano). Avoid narrow toe box!

**4. If Flatfoot + Hallux Valgus Coexist, Is Treatment Different?**

- Surgical planning: If flatfoot is not corrected, relapse risk increases with only hallux valgus surgery.
- Combined procedures: Lateral column lengthening (for flatfoot). Scarf osteotomy (for hallux valgus).

**Patient Experience:**

- In those who start using insoles in the early period (ages 10–20), deformity progression is 70% less.
- Exercises targeting the tibialis posterior tendon are critical after age 40.

**Important Warning:**

Flatfoot alone does not cause hallux valgus, but the risk explodes with high heels/narrow shoes and genetics.

**Conclusion:**

Flatfoot is an important predisposing factor for hallux valgus, but risk can be controlled with correct precautions.

### **Is hallux valgus seen in children?**

Yes, it can be seen in children too! However, it appears for different reasons than in adults and is generally associated with genetic/flexible foot structure.

**Causes of Hallux Valgus in Children**

**1. Genetic Predisposition (Most Common Cause)**

- If there is a family history (especially in mother or father), the risk in the child increases by 30–50%.
- It is commonly seen in children with hypermobility syndrome due to connective tissue laxity.

**2. Incorrect Footwear Choice**

- Narrow/pointed-toe shoes can deform the still-flexible bones of children.
- Shoes that are too small for the foot compress the great toe.

**3. Flatfoot (Pes Planus)**

- Childhood flatfoot increases the risk of hallux valgus by causing excessive pronation.

**4. Neuromuscular Diseases**

- Conditions such as cerebral palsy, Down syndrome cause deformity due to muscle imbalance.

**Symptoms of Hallux Valgus in Children**

- Mild curvature of the great toe (may be painless in early period).
- Discomfort or pressure marks on the inner side while wearing shoes.
- Inward rolling (pronation) while walking.

**When to Be Concerned?**

- Curvatures becoming prominent between ages 8–14 may be progressive.
- If there is pain, redness, or callus, consult a pediatric orthopedic specialist immediately.

**Treatment and Precautions**

**1. Non-Surgical Methods (Effective in Early Period)**

- Orthopedic shoe: Wide-toed models with medial arch support.
- Night splint: Helps correct curvature in children during the growth period.
- Exercises: Movements strengthening great toe muscles (towel grab, marble pick-up).

**2. Surgical Treatment (Rarely Needed)**

- Only considered in severe pain/progressive deformity.
- If bone growth is not complete (approximately age 14 for girls, 16 for boys), special techniques are applied.

**Critical Warnings for Parents**

- When buying shoes: There should be 1 cm space between the great toe and the tip of the shoe.
- Sport selection: Be careful in activities that strain the feet such as ballet/gymnastics.
- Follow-up: Have a foot scan done once a year.

**In Summary:**

- Hallux valgus is not uncommon in children, but can be controlled with early precautions.
- If it runs in the family or curvature is noticed, orthopedic check-up every 6–12 months is essential!

### **Is pain, swelling, and redness normal in hallux valgus?**

Pain is normal, but if swelling and redness are present, caution is needed! These symptoms may indicate that the deformity is progressing or that complications such as infection/bursitis are beginning.

**1. Pain (Most Common Complaint)**

- Why Does It Occur? Friction of the bony prominence against the shoe ('bunion'). Joint degeneration (beginning of arthritis).
- When Is It Normal? Temporary pain after long walks/tight shoes.
- When Is It Abnormal? Pain continuing even at rest (may require surgery).

**Solution:**

- Ice application (15 minutes, 2–3 times a day).
- NSAIDs (ibuprofen).
- Use silicone bunion protector.

**2. Swelling**

- Why Does It Occur? Chronic irritation → Bursitis (swelling of the joint sac). Post-trauma edema.
- Is It Dangerous? Red-warm swelling may be a sign of infection!

**Solution:**

- Elevate the feet.
- Prevent impact (wear wide shoes).

**3. Redness**

- Why Does It Occur? Infection (risk of cellulitis!). Severe friction.

**Emergency Symptoms:**

- Fever, chills → Go to the emergency room!

**Solution:**

- Hygiene: Don't walk barefoot.
- Antibiotic cream (with physician's recommendation).

**When Should You Go to the Doctor?**

- If pain wakes you at night,
- If swelling lasts more than 3 days,
- If redness is spreading,
- If accompanied by fever.

**Precautions**

- Shoes: Prefer soft, leather, and wide-toed.
- Ice: Twice a day for swelling.
- Exercise: Do great toe stretching movements.

## **2. Treatment Options and Surgical Decision-Making (DeepSeek)**

### **Is non-surgical treatment possible in hallux valgus? (DeepSeek)**

Yes, non-surgical treatment is possible in early and moderate stage hallux valgus! However, it does not completely eliminate the bony protrusion; it stops progression and reduces pain.

**Non-Surgical Treatment Options**

**1. Correct Footwear Choice (Most Critical Step!)**

- Wide-toed, soft-soled shoes (e.g.: Hoka, Altra, Birkenstock).
- Definitely avoid high-heeled and narrow shoes!
- Orthopedic insole (custom-made with medial arch support).

**2. Silicone or Gel Protectors**

- Bunion splint (night splint): Keeps the great toe in a straight position.
- Interdigital spacer: Reduces pressure by separating the great toe from the index finger.

**3. Physical Therapy and Exercises**

- Towel curl exercise: Pulling a towel placed under the foot with the toes.
- Toe spread (toe spreading): Spreading the toes as much as possible and holding for 5 seconds.
- Ankle and plantar fascia stretching: Reduces flatfoot effect.

**4. Pain and Inflammation Management**

- Ice application: 2–3 times a day for 15 minutes.
- NSAID medications (ibuprofen, naproxen).
- Cortisone injection: If severe bursitis is present (under physician supervision).

**5. Weight Loss and Activity Modification**

- Excess weight increases the load on the feet.
- Prefer swimming/cycling instead of high-impact sports (running, jumping).

**How Effective Is Non-Surgical Treatment?**

| **Stage** | **Treatment Success** |
| --- | --- |
| Early Stage (Mild curvature) | 70–80% success (can completely resolve pain). |
| Moderate Stage (Prominent protrusion) | 40–50% (reduces pain, can stop progression). |
| Advanced Stage (Severe deformity) | Surgery may be needed. |

**When Is Surgery Needed?**

- Pain even at rest,
- Great toe pushing other toes (cross-toe deformity),
- Making shoe wearing impossible.

**Conclusion:**

- Non-surgical methods are very effective in the early period!
- If pain does not resolve despite 6 months of regular treatment, evaluate surgical options.

### **Do methods such as orthopedic insoles, splints, pads, exercises, or taping work in hallux valgus? (DeepSeek)**

Yes, these methods work in early and moderate stage hallux valgus! However, they do not eliminate the bony protrusion; they reduce pain and slow progression. Here are details supported by scientific evidence:

**1. Orthopedic Insoles**

- Corrects flatfoot/pronation → reduces load on the great toe.
- Relieves pain by 40–60% (Journal of Foot and Ankle Research).
- Which type of insole? Medial arch support (carbon fiber or thermoplastic). Custom-made (should be individually adjusted by a podologist).
- Effective? Very beneficial in early stage, only provides support in advanced stage.

**2. Night Splint (Bunion Splint)**

- Slows deformity progression by keeping the great toe in neutral position.
- Effective in children and early stage adults.
- Effective? Can reduce the curvature angle by 3–5 degrees with 6 months of regular use (Foot & Ankle International). Insufficient alone in advanced stage.

**3. Silicone Pad and Interdigital Spacer**

- Prevents callus and pain by reducing friction.
- Interdigital spacer helps correct the great toe.
- Effective? Reduces pain by 50–70%, but does not correct deformity. Best results when used inside shoe.

**4. Exercises**

**3 Most Effective Exercises:**

- Towel Curl: Pulling a towel placed under the foot with the toes.
- Toe Spread: Spreading fingers as much as possible and holding for 5 seconds.
- Marble Pick-Up: Grasping and lifting small balls/stones with the toes.
- Effective? If done regularly, strengthens great toe muscles and increases flexibility. Insufficient alone, should be combined with other methods.

**5. Kinesio Taping**

- Helps maintain the great toe in a straighter position.
- Provides temporary support in athletes.
- Effective? Provides immediate relief, but no permanent effect. Long-term use can cause skin irritation.

**Which Method Works When?**

| **Method** | **Early Stage** | **Moderate Stage** | **Advanced Stage** |
| --- | --- | --- | --- |
| Orthopedic insole | ✔️✔️✔️ | ✔️✔️ | ✔️ |
| Night splint | ✔️✔️✔️ | ✔️✔️ | ❌ |
| Silicone pad | ✔️✔️ | ✔️ | ✔️ |
| Exercises | ✔️✔️ | ✔️ | ❌ |
| Taping | ✔️ | ❌ | ❌ |

**Conclusion: For Which Patients Is It Appropriate?**

- Early stage (mild curvature, occasional pain) → Can be controlled with insoles + exercise + pad.
- Moderate stage (prominent protrusion, frequent pain) → Night splint + insole combination should be tried.
- Advanced stage (severe deformity, constant pain) → Surgery may be the only permanent solution.

Extra tip: If you don't correct your shoe choice, none of these methods alone will be sufficient!

### **In which cases is surgery necessary for hallux valgus? (DeepSeek)**

Surgery may be needed in the following situations:

- If pain prevents daily life (inability to wear shoes, nighttime pain).
- If deformity is progressing (if great toe is pushing other toes).
- If non-surgical treatments (insoles, splints) have not worked.

**1. Factors Affecting the Surgical Decision**

**A) Pain and Functional Loss**

- Pain even at rest.
- Shortening of walking distance (e.g., severe pain after 500 meters).
- Preventing sports/work.

**B) Severity of Anatomical Deformity**

- HV angle >20° (should normally be <15°).
- Great toe sliding under/over the 2nd toe (cross-toe deformity).
- Bony protrusion making shoe wearing impossible.

**C) Joint Damage (Arthrosis)**

- Calcification seen on X-ray.
- Decrease in joint range of motion (feeling of stiffness).

**2. Which Type of Surgery Is Applied?**

| **Degree of Deformity** | **Type of Surgery** | **Explanation** |
| --- | --- | --- |
| Mild-Moderate | Chevron osteotomy | Bone is cut and corrected. |
| Severe | Scarf osteotomy | More complex bone sliding. |
| With Arthritis | Joint fusion (arthrodesis) | Calcified joint is stabilized. |
| Very Advanced | Prosthesis (artificial joint) | Rarely preferred. |

**3. Postoperative Process**

- 1–2 days: Crutches may be needed.
- 2–6 weeks: Special shoe (post-op shoe) is worn.
- After 3 months: Can return to normal shoes.
- Full recovery: 6–12 months (return to sports).

**Caution!**

- Relapse risk (5–15%), especially higher in young women and if flatfoot coexists.
- Correct surgeon selection is very important (experienced orthopedist essential).

**4. What Happens If I Don't Have Surgery?**

- Pain increases, walking becomes difficult.
- Deformity (hammer toe) may develop in the 2nd toe.
- Calcification progresses in the joint.

**Critical Questions for Final Decision**

- 'Can't I step on my foot even with pain relievers?'
- 'Is wearing shoes a nightmare for me?'
- 'Is my great toe pushing other toes?'

If your answer is 'YES,' surgery time may have come.

Additional Information: Before surgery, definitely get a 2nd expert opinion. Although laser treatment for hallux valgus may be offered in some cases, the scientific evidence for these is weak.

### **What are the types of hallux valgus surgery? (DeepSeek)**

**Hallux Valgus Surgery Types**

Hallux valgus surgeries are determined according to severity of deformity and joint damage. Here are the most commonly used 10 surgical methods and which conditions they are preferred in:

**1. For Mild-Moderate Deformities**

**A) Chevron Osteotomy**

- For whom? HV angle <30°, those with intact joint.
- How? Bone is cut in V shape and shifted.
- Advantage: Fast recovery (4–6 weeks).

**B) Scarf Osteotomy**

- For whom? Moderate deformity + flatfoot.
- How? Bone is cut in Z shape and lengthened.
- Advantage: More stable, lower relapse risk.

**C) Akin Osteotomy**

- For whom? Curvature in great toe joint (combined with chevron).
- How? A small piece is removed from the tip of the bone.

**2. For Severe Deformities**

**D) Lapidus Arthrodesis**

- For whom? Advanced HV + flatfoot.
- How? Metatarsal bone–ankle joint is stabilized.
- Advantage: Method with lowest relapse rate (5%).

**E) Proximal Metatarsal Osteotomy**

- For whom? Those with HV angle >40°.
- How? Cut is made from the upper part of the bone.

**3. If Joint Damage Is Present**

**F) Arthrodesis (Joint Fusion)**

- For whom? Those with calcification (arthrosis) developed.
- How? Joint movement is completely removed.
- Advantage: Completely relieves pain.

**4. Minimally Invasive (Closed) Methods**

**H) PERCUTANEOUS (Closed) Osteotomy**

- For whom? Early stage HV, small protrusions.
- How? Bone is corrected through small holes from the skin.
- Advantage: No scar, fast recovery.

**I) Laser Hallux Valgus Treatment**

- Is it real? No! It has no scientific basis.

**5. For Special Cases**

**J) McBride Soft Tissue Balancing**

- For whom? Young patients, ligament laxity.
- How? Tendons are repositioned.

**Which Surgery Is Preferred When?**

| **Deformity Type** | **Preferred Surgery** |
| --- | --- |
| Mild (HV angle <20°) | Chevron, PERCUTANEOUS |
| Moderate (HV 20–40°) | Scarf, Akin |
| Severe (HV >40°) | Lapidus, Proximal Osteotomy |
| With Arthritis | Arthrodesis |

**Postoperative**

- Week 1: Cast/special shoe.
- Week 6: Light walking.
- Month 3: Normal shoes.

Note: Relapse rate 5–15% (especially in young women). Correct surgeon selection is the most critical factor!

### **What should I do if my child has the onset of hallux valgus? (DeepSeek)**

**What You Need to Do If Your Child Has the Onset of Hallux Valgus**

**1. First Go to an Orthopedic Specialist for Correct Diagnosis**

- Should be examined by a pediatric orthopedist.
- Curvature angle (HV angle) should be measured with X-ray.

**2. Immediately Change Shoe Selection**

- Completely abandon narrow, hard, or heeled shoes.
- Preferred Shoe Features: Wide and round toe (not pressing on great toe). Flexible sole (not rigid, can bend). Supported insole (custom-made if flatfoot exists).

**3. Definitely Use Insoles If Flatfoot Exists**

- Orthopedic insole with medial arch support is essential.
- Get it with the recommendation of a podologist or orthopedic specialist.

**4. Use Night Splint (Bunion Splint)**

- Keeps the great toe in a straight position, slows deformity progression.
- 8–12 hours of nighttime use is recommended.

**5. Do Exercises**

- Toe Spread: Move the great toe away from other toes.
- Towel Curl: Pull towel placed under foot with toes.
- Marble Picking: Grasp and lift small balls with foot toes.

**6. Be Careful in Sport Selection**

- Be careful in sports that strain the feet such as ballet, gymnastics, football.
- Low-impact sports such as swimming and cycling are more appropriate.

**7. Regular Follow-up Is Essential!**

- Go to orthopedic check-up every 6 months.
- Early intervention is very important if curvature is increasing.

**Is Surgery Needed?**

- Surgery is very rare in children!
- Only if: Pain wakes at night, Pushing other toes, Progressing despite all precautions, surgery is considered.

**In Summary:**

- With early diagnosis + correct footwear + exercises, progression can be stopped in most children.
- Don't say 'It will pass'! Untreated hallux valgus leads to serious problems in adulthood.

## **3. Postoperative Course and Functional Recovery (DeepSeek)**

### **How long does recovery take after hallux valgus surgery? (DeepSeek)**

Full recovery takes 3–6 months, but return to daily life begins within 4–6 weeks. Here is the detailed recovery schedule:

**1. First Week After Surgery**

- Pain & Swelling: Severe swelling and pain may occur in the first 3 days (use ice + analgesics). Foot should be kept elevated (reduces swelling).
- Walking: Crutch support may be needed with special post-op shoe or cast. Walk without full weight bearing on foot.

**2. Weeks 2–6**

- Check-ups: Sutures are removed, bone position is checked with X-ray.
- Walking: From the 4th week, gradual weight bearing with special shoe. Car driving: If right foot surgery, wait 4–6 weeks.
- Physical Therapy: Great toe motion exercises begin (passive stretching).

**3. Weeks 6–12**

- Return to Shoes: At week 6, wide-toed sports shoe can be worn. Heeled/narrow shoe requires waiting 3 months.
- Activity: Swimming/cycling: After the 6th week. Running: After the 3rd month.

**4. After 3–6 Months**

- Full Recovery: Bone healing is completed (checked with X-ray). Can return to all sports at 6 months (football, tennis, etc.).
- Caution: Mild swelling in the surgical area can last 1 year.

**5 Critical Rules to Speed Recovery**

- Don't smoke (delays healing).
- Regularly use the post-op shoe given by the physician.
- Apply ice (first 2 weeks 3–4 times a day).
- Eat high in protein (speeds bone repair).
- Don't miss physical therapy exercises.

**When Should You Urgently Consult a Physician?**

- Fever or discharge of pus from surgical site (sign of infection).
- Sudden severe pain (suspicion of fracture/dislocation).
- Bruising/numbness in foot (circulatory disorder).

**Summary Table: Recovery After Hallux Valgus Surgery**

| **Period** | **What Can Be Done** | **Prohibited** |
| --- | --- | --- |
| 0–2 Weeks | Walking with crutches, ice | Loading foot, wearing shoes |
| 3–6 Weeks | Walking with post-op shoe | High-heeled narrow shoes, sports |
| 6–12 Weeks | Sports shoe, swimming | Running, jumping |
| 3–6 Months | Return to all activities | Overly strenuous sports |

- 'When can I return to work after surgery?' Office work: 2–3 weeks later. Standing work: 6–8 weeks later.
- 'Will there be a scar?' With minimally invasive methods, scars are nearly invisible.
- 'Will it recur?' 5–10% risk, especially in young women.

Be patient after surgery! The first 2 weeks may be difficult, but after 6 months you will be able to walk without pain.

### **When can I wear normal shoes after hallux valgus surgery? (DeepSeek)**

After 6–8 weeks you can wear comfortable sports shoes, but for narrow/heeled shoes you should wait 3–6 months.

**Detailed Shoe Transition Schedule**

**1. First 2 Weeks**

- Special post-op shoe (open front, rigid sole) is worn.
- Normal shoes FORBIDDEN!

**2. Weeks 3–6**

- Wide-toed sports shoe (e.g.: Hoka, Altra) can be worn.
- Use of insole may be needed.

**3. Weeks 6–8**

- Most patients transition to normal shoes (but not narrow-toed ones). Recommended models: New Balance 990v5 (wide toe), Skechers Arch Fit (supported sole).

**4. After 3–6 Months**

- Heeled/narrow shoes can be worn, but: Don't wear more than 2–3 hours a day. Use silicone bunion protector.

**What Shoe Should Be Worn When?**

| **Shoe Type** | **Wearing Time** | **Points to Note** |
| --- | --- | --- |
| Post-op shoe | 0–6th week | Don't remove until your physician allows |
| Sports shoe | 6th week | Should be wide-toed, flexible-soled |
| Daily shoe | 8th week | Add orthopedic insole |
| Heeled shoe | 3–6th month | Shouldn't be higher than 5 cm |

**5 Golden Rules When Choosing Shoes**

- Toe test: Great toe should not touch the tip of the shoe.
- Flexibility: You should be able to bend the sole with your hand.
- Sole support: Arch (heel) area should be rigid.
- Material: Prefer breathable leather/fabric.
- Shopping time: Buy in the evening hours (feet are swollen).

**Important Warnings!**

- If you wear high heels before 6 months: Relapse risk increases!
- If pain occurs: Immediately remove narrow shoes.

**Shoe Recommendations**

- Sports: Hoka Bondi, Brooks Ghost
- Daily: Ecco Soft 7, Clarks Unstructured
- Office: Naturalizer Marianne, Rockport Total Motion

When buying your first normal shoe after surgery, definitely take your orthopedic insole with you! Note: The shoe transition process varies from person to person. Definitely follow your physician's recommendations.

### **Will there be a scar after hallux valgus surgery? (DeepSeek)**

Yes, scars may remain, but for most patients they are not prominent. The visibility of scars depends on the surgical technique, your skin structure, and the healing process.

**1. Scar Status According to Surgical Technique**

**A) Open Surgery (Classic Methods)**

- Scar: 3–5 cm in length, in the form of a thin line on the medial surface of the foot.
- Appearance: Fades over time but does not completely disappear.
- Examples: Chevron, Scarf, Lapidus surgeries.

**B) Minimally Invasive (Closed) Surgery**

- Scar: Small holes of 1–2 mm (nearly invisible).
- Appearance: Becomes indistinct within 6–12 months.
- Examples: PERCUTANEOUS osteotomy.

**C) Is It Done with Laser?**

- Reality: Laser hallux valgus surgery is not a scientifically proven method.

**2. Factors Affecting Scar Appearance**

- Skin Type: Risk of hyperpigmentation (dark-colored scar) increases in those with darker skin.
- Wound Care: Early suture removal or infection can thicken the scar.
- Genetics: Tendency to keloid formation.

**3. Five Critical Recommendations to Minimize Scar**

- First 2 Weeks After Surgery: Keep the wound site dry, don't let water touch. Regularly apply the antibiotic cream given by the physician.
- For 3–6 Months: Use silicone scar gel (e.g.: Dermatix, Kelo-cote). Apply sunscreen (SPF 50+) (sun makes scar darker).
- Massage: From the 6th week, do gentle circular massage (softens scar tissue).
- Nutrition: Eat high in vitamin C and protein (increases collagen production).
- Don't smoke: Nicotine slows healing and causes scar to become more prominent.

**Summary Table: Scar Status Comparison**

| **Surgery Type** | **Scar Size** | **Prominence** | **Does It Completely Disappear?** |
| --- | --- | --- | --- |
| Open surgery | 3–5 cm | Moderate | No (fades) |
| Minimally invasive | 1–2 mm | Very little | Nearly disappears in 1 year |
| PERCUTANEOUS | Negligible | None | Yes |

'What can I do if the scar bothers me?' Laser treatment (for red scars). Dermaroller (increases collagen production). 'My surgical scar looks ugly, is aesthetic improvement possible?' You can get a plastic surgery consultation (scar revision).

Final Word: A surgical scar may be a small price to pay for getting rid of hallux valgus. However, it can be kept to a minimum with proper care.

### **How long does it take to return to daily life and work after hallux valgus surgery? (DeepSeek)**

- Office work: You can return after 2–3 weeks.
- Standing work: You should wait 6–8 weeks.
- Full recovery and sports: Takes 3–6 months.

**Detailed Recovery Schedule**

**1. First 2 Weeks (Early Recovery Period)**

- Weight bearing: Partial weight bearing with crutches or special post-op shoe.
- Return to work: Those with office work can work from home.
- Activity: Bed rest + ice application important.

**2. Weeks 3–6 (Intermediate Recovery Period)**

- Return to office work: From the 3rd week (but keep your foot elevated).
- Those with standing work: Should take leave until the 6th week (teacher, nurse, waiter, etc.).
- Car driving: If right foot surgery, wait 4–6 weeks.

**3. Weeks 6–12 (Advanced Recovery Period)**

- Standing work: Can return to full shift after the 8th week.
- Sports: Swimming/cycling from the 6th week, running from the 3rd month.

**4. After 3–6 Months (Full Recovery)**

- Heavy sports: Activities such as football and tennis should be done after 6 months.
- Heeled shoes: Should not be worn before the 4th month.

**Factors Affecting Return to Work**

| **Factor** | **Does It Affect Return to Work Time?** |
| --- | --- |
| Surgical technique (open vs. closed) | Yes (closed surgeries are faster) |
| Physical difficulty of work | Yes (standing workers return later) |
| Person's recovery rate | Yes (may be delayed in smokers) |
| Complications (infection, etc.) | Yes (may delay 1–2 weeks) |

**5 Tips for Those Who Want to Return to Work Early**

- Make a flexible work plan with your physician (part-time or remote work).
- Keep your foot elevated at the office (reduces swelling).
- Footwear choice: Use wide-toed and orthopedic insoles.
- Apply ice during breaks (especially the first 1 month).
- Don't miss physical therapy (prevents strength loss).

**Which Professions Return When?**

| **Profession** | **Return to Work Time** | **Recommendations** |
| --- | --- | --- |
| Computer work | 2–3 weeks | Use foot support |
| Teacher | 6–8 weeks | Increase rest breaks |
| Healthcare worker | 8 weeks | Wear compression stockings |
| Driver | 6 weeks (manual transmission) | Wait 8 weeks for long journeys |
| Athlete | 3–6 months | Physical therapy essential! |

**Caution! Early Return Risks**

- Increased pain,
- Delayed healing,
- Recurrence of deformity.

**In Summary:**

- Light work: 2–3 weeks,
- Standing work: 6–8 weeks,
- Heavy physical work: 3 months+.

The first 2 weeks after surgery are very critical! Focus on healing; don't rush to return to work.

### **Does hallux valgus recur after surgery? (DeepSeek)**

Yes, it can recur! The relapse rate is between 5–15%. However, with the correct surgical technique and patient compliance, this risk can be reduced below 5%.

**Relapse Causes**

**1. Patient-Related Factors**

- Genetic predisposition (risk increases if there is family history).
- Non-compliance with postoperative rules (wearing narrow shoes early, excessive loading).
- If flatfoot is not treated, relapse risk doubles.

**2. Surgery-Related Factors**

- Insufficient correction (if HV angle is not fully corrected).
- Incorrect technique selection (performing simple osteotomy in severe deformity).

**3. Anatomical Factors**

- Hypermobility (excessive joint flexibility).
- Metatarsus primus varus (excessive angulation of the 1st metatarsal bone).

**What Is the Relapse Risk in Each Technique?**

| **Surgery Type** | **Relapse Rate** | **Reason** |
| --- | --- | --- |
| Chevron osteotomy | 5–10% | Good results in mild deformities |
| Scarf osteotomy | 5–8% | Moderately stable |
| Lapidus arthrodesis | <5% | Most stable method |
| Percutaneous (closed) | 8–12% | Experienced surgeon required |

**7 Golden Rules to Prevent Relapse**

- Don't wear narrow/heeled shoes for 6 months after surgery.
- Use orthopedic insoles (essential if flatfoot exists!).
- Regular check-ups (have X-rays at 3rd-6th-12th months).
- Do great toe exercises (toe spread, towel curl).
- Get rid of excess weight (reduces foot load).
- Don't smoke (delays bone healing).
- Choose the right surgeon (experienced orthopedist essential).

**Relapse Symptoms**

- Start of new curvature in the great toe,
- Return of pain,
- New discomfort when wearing shoes.

**What Is Done If Relapse Occurs?**

- In early stage → Night splint + physical therapy can be tried.
- In advanced stage → Revision surgery required (more stable techniques such as Lapidus are preferred).

**Important Statistics**

- Relapse risk is higher in young women (hormones + ligament laxity).
- If flatfoot coexists, relapse rate can reach 20%.
- In those who comply with rules for 1 year after surgery, risk is <3%.

**Conclusion:**

Surgical success depends not only on the surgeon, but also on you! If you comply with the rules, relapse risk is minimal.

Additional information: To prevent relapse, hyaluronic acid injections (increases joint lubrication) or PRP treatment can be tried. Consult your physician!

## **4. Long-Term Outcomes and Quality of Life (DeepSeek)**

### **Does hallux valgus worsen over time if left untreated? (DeepSeek)**

Yes, hallux valgus (bunion) is generally a progressive deformity that worsens over time. If left untreated, the following situations may occur:

**Reasons for Worsening Over Time:**

- Pressure and Improper Footwear Use: Tight, narrow, or high-heeled shoes increase deformity. Disruption of foot mechanics leads to the protrusion growing.
- Progressive Deformity: The great toe (1st toe) gradually slides under or over the 2nd toe. Collapse of the foot arch and flatfoot can develop.
- Joint and Soft Tissue Degeneration: Arthrosis (calcification) may develop in the joint. Tendon imbalance increases complaints.
- Pain and Functional Loss: Pain gradually intensifies, walking becomes difficult. Secondary problems such as calluses and bursitis may develop.

**When Should You Consult a Physician?**

- If pain is preventing daily activities,
- If the protrusion is rapidly growing,
- If wearing shoes has become difficult,
- If toe deformity (distortion) has begun.

**Treatment Options:**

- Early stage: Orthoses, correct footwear, exercises.
- Advanced cases: Surgery (osteotomy, fusion, etc.).

Conclusion: Hallux valgus generally does not improve on its own; early intervention is important. If you have complaints, consult an orthopedic specialist.

### **Does hallux valgus affect other toes? (DeepSeek)**

Yes, hallux valgus (bunion) can also adversely affect other toes over time, causing various deformities and painful conditions. Here are the main effects:

**1. Deformities in Other Toes**

- Hammer Toe: The great toe shifting outward causes pressure on the 2nd toe, causing it to rise upward or deform. Over time, the toe joints stiffen and become painful.
- Claw Toe: Incorrect alignment of the great toe can cause other toes to curl and take on a claw-like appearance.
- Crossover Toe: In advanced hallux valgus, the 2nd toe can cross over or under the great toe.

**2. Calluses and Pressure Sores**

- The incorrect position of the great toe creates excessive pressure on other toes and the sole of the foot.
- This condition can lead to calluses, blisters, and painful bursitis.

**3. Disruption of Foot Mechanics**

- Hallux valgus disrupts the foot arch and walking pattern, causing: Metatarsalgia (pain in the forefoot), Flatfoot, Tendon problems.

**4. What Happens If Left Untreated?**

- As deformity progresses, wearing shoes becomes difficult,
- Chronic pain may develop,
- Walking and balance may be disrupted.

**What Should Be Done?**

- Early period: Special footwear, interdigital silicones, exercises.
- Advanced cases: Surgical intervention may be needed.

Conclusion: Hallux valgus can disrupt the entire foot structure, not just the great toe. If you have complaints, consulting an orthopedic or foot surgery specialist is recommended.

### **Does hallux valgus affect my walking or playing sports? (DeepSeek)**

Yes, hallux valgus can significantly affect your walking and sports performance over time. Here are possible effects and their causes:

**1. Effects on Walking**

- Pain and Discomfort: Pressure in the protrusion area causes pain especially during long walks. If calluses or bursitis are present, every step can be uncomfortable.
- Mechanical Changes: Push power of the great toe decreases, disrupting walking pattern (biomechanics). Body weight shifts to other toes, metatarsalgia (forefoot pain) may develop.
- Balance Problems: As deformity progresses, foot stability decreases and balance loss may occur.

**2. Effects on Sports Performance**

- Running and High-Impact Sports: Running on hard surfaces, jumping, etc. intensify pain. Due to reduced push power of the great toe, speed and performance may decrease.
- Stress on Ankle and Knee: Incorrect weight distribution can cause ankle, knee, and even hip pain.
- Flexibility and Movement Restriction: Hallux valgus restricts great toe dorsiflexion (upward movement), making some sports movements difficult (e.g., squat, lunge).

**3. What Can Be Done?**

- Correct Footwear Choice: Use wide-toed, supportive-soled sports shoes. Avoid high-heeled or narrow shoes.
- Special Supports: Orthoses (hallux valgus splints) or insoles can be used.
- Exercises: Do exercises that strengthen and stretch foot toes.
- Early Treatment: If pain and deformity progress, surgical options should be evaluated.

**Conclusion:**

Hallux valgus can gradually disrupt your walking and sports ability. Early intervention can slow progression and help maintain your quality of life. If you have complaints, consult an orthopedic specialist.

### **Does losing weight reduce hallux valgus-related complaints? (DeepSeek)**

Yes, losing weight can help reduce pain and slow the progression of hallux valgus. Here is a detailed explanation:

**1. Relationship Between Weight and Hallux Valgus**

- Increasing Load on the Feet: Excess weight places more pressure on the feet, increasing stress on the great toe joint. This can accelerate protrusion growth and intensify pain.
- Disrupts Walking Mechanics: Weight excess can lead to collapse of the foot arch and flatfoot, which can worsen hallux valgus.

**2. What Changes When Weight Is Lost?**

- Reduction in Pain: When the load on the feet decreases, pressure on the great toe joint reduces and pain lessens.
- Deformity Progression Slows: Less mechanical stress reduces the speed of deformation of bone and joint.
- Wearing Shoes Becomes Easier: With weight loss, edema in the feet may also decrease, providing comfort inside shoes.

**3. Is Losing Weight Alone Sufficient?**

- No, losing weight can relieve complaints but does not completely correct the anatomical deformity of hallux valgus.
- Should Be Combined with Supportive Treatments: Correct footwear choice (wide-toed, supportive-soled), Hallux valgus splints or insoles, Exercises strengthening foot muscles (e.g., pulling towel with toes, foot arch exercises).
- Surgery May Be Needed in Advanced Cases: If deformity has progressed too much, weight loss alone may not be sufficient; surgery may be required.

**4. Recommendations for Losing Weight (Hallux Valgus Friendly)**

- Low-Impact Exercises: Prefer activities that do not place excessive load on the feet such as swimming, cycling, yoga. Walk instead of running (but with appropriate footwear).
- Healthy Nutrition: Limit salt to reduce edema and inflammation; consume antioxidant foods.
- Pay Attention to Foot Health: Avoid movements that will strain your foot while losing weight.

**Conclusion:**

Losing weight is effective in slowing pain and progression caused by hallux valgus, but is not a solution by itself. Better results are obtained when combined with supportive treatments. If complaints continue, consult an orthopedic specialist.

Note: Losing weight will also be beneficial not only for foot problems but for your general health.
