# Supplementary material for: Evaluating the reliability, quality, and readability of AI-generated patient education on hallux valgus: a comparative study of large language models
Source: BMC Med Inform Decis Mak. 2026 Jul 30;26:433. doi: 10.1186/s12911-026-03722-8 (PMC13711935; doi:10.1186/s12911-026-03722-8)
Supplement: Supplementary file 2 [file 12911_2026_3722_MOESM2_ESM.docx]

**Supplementary Table S1. Scoring anchors used for expert evaluation of AI-generated responses**

| **Score** | **Reliability Scale Interpretation** | **Usefulness Scale Interpretation** |
| --- | --- | --- |
| 1 | Very unreliable: Most information could not be verified with scientific medical sources and contained major inaccuracies or omissions. | Not useful: The response was difficult to understand, contained major deficiencies, and was unlikely to assist patient understanding. |
| 2 | Poor reliability: Considerable portions of the response were inaccurate, incomplete, or unsupported by current medical evidence. | Minimally useful: Some understandable information was provided, but important deficiencies limited practical patient value. |
| 3 | Limited reliability: Much of the information was generally verifiable, although important inaccuracies or omissions remained. | Limited usefulness: Basic information was presented, although important details remained incomplete or unclear. |
| 4 | Moderate reliability: Most information was scientifically accurate, with minor inaccuracies or incomplete details. | Moderately useful: The response provided understandable and partially practical information for patients despite some limitations. |
| 5 | Good reliability: The majority of information was accurate, evidence-based, and clinically appropriate, with only minimal deficiencies. | Useful: Most clinically relevant information was presented clearly and could support patient understanding. |
| 6 | Very good reliability: Information was highly accurate, comprehensive, and largely consistent with current medical knowledge. | Very useful: The response was clear, comprehensive, and practically informative for patients. |
| 7 | Excellent reliability: Information was consistently accurate, comprehensive, and clinically appropriate, without any major inaccuracies identified during expert review. | Extremely useful: The response was highly understandable, comprehensive, and provided substantial educational value for patients. |

**Supplementary Table S2. Mixed-effects sensitivity analyses accounting for clustering at the question level**

| **Outcome** | **Comparison (Reference = ChatGPT-4o)** | **β Coefficient** | **95% CI** | **p-value** |
| --- | --- | --- | --- | --- |
| **Reliability** | Gemini-2.5-Flash vs. ChatGPT-4o | 0.667 | 0.487 to 0.846 | <0.001 |
|  | DeepSeek-V3 vs. ChatGPT-4o | -0.117 | -0.296 to 0.063 | 0.204 |
| **Usefulness** | Gemini-2.5-Flash vs. ChatGPT-4o | 0.217 | -0.022 to 0.456 | 0.076 |
|  | DeepSeek-V3 vs. ChatGPT-4o | 0.300 | 0.061 to 0.539 | 0.014 |
| **Global Quality Score (GQS)** | Gemini-2.5-Flash vs. ChatGPT-4o | 0.000 | -0.228 to 0.228 | 1.000 |
|  | DeepSeek-V3 vs. ChatGPT-4o | -0.067 | -0.294 to 0.161 | 0.566 |

**Abbreviations:** β, regression coefficient; CI, confidence interval; GQS, Global Quality Score.

Sensitivity analyses were performed using mixed-effects linear regression models treating the ordinal rating scales as approximately continuous variables, with clinical question included as a random effect.


**Supplementary Table S3. Representative examples of potentially oversimplified or misleading AI-generated responses identified during qualitative expert review**

| **Clinical Question** | **AI Model** | **Representative Response Excerpt** | **Potential Concern Identified by Reviewers** | **Clinical Interpretation** |
| --- | --- | --- | --- | --- |
| “Do methods such as orthopedic insoles, splints, pads, exercises, or taping work?” | DeepSeek-V3 | “Early stage (mild curvature) → 70–80% success” | Quantitative success estimates were presented without supporting evidence or clarification of outcome definitions. | Conservative treatment may improve symptoms and footwear tolerance but does not reliably correct deformity progression. |
| “Is non-surgical treatment possible in hallux valgus?” | DeepSeek-V3 | “Early stage … can completely resolve pain.” | Potentially optimistic wording may create unrealistic expectations regarding conservative management. | Symptom relief is possible in selected patients; however, treatment response is variable and structural correction is generally limited without surgery. |
| “Does hallux valgus recur after surgery?” | ChatGPT-4o | “Recurrence risk is lower with minimally invasive methods and appropriate post-op care.” | The statement may oversimplify recurrence risk and imply superiority of minimally invasive techniques without adequate clinical context. | Recurrence risk depends on multiple factors including deformity severity, surgical technique selection, patient-related factors, and postoperative compliance. |
| “What are the types of hallux valgus surgery?” | DeepSeek-V3 | “Laser hallux valgus treatment … has no scientific basis.” | The statement was considered overly absolute and lacked nuance regarding minimally invasive surgical marketing terminology. | Evidence supporting isolated “laser” hallux valgus treatment remains limited, although minimally invasive surgical techniques are increasingly used in selected patients. |
| “When can I wear normal shoes after surgery?” | DeepSeek-V3 | “After 6–8 weeks you can wear comfortable sports shoes.” | Recovery timelines were sometimes presented as fixed schedules despite known variability between patients and surgical procedures. | Return to footwear varies according to surgical technique, bone healing, swelling, and surgeon preference. |
| “Is pain, swelling, and redness normal in hallux valgus?” | DeepSeek-V3 | “Red-warm swelling may be a sign of infection!” | Some responses used alarm-oriented language that could increase unnecessary patient anxiety in otherwise common inflammatory presentations. | Pain, swelling, and local irritation are common in hallux valgus; however, rapidly progressive redness, fever, or severe symptoms require clinical assessment. |

**Supplementary Table 4.** **Clinical takeaways.**

| **Domain** | **Highest-scoring model** | **Key finding** | **Clinical recommendation** |
| --- | --- | --- | --- |
| Reliability | Gemini-2.5-Flash | Significantly higher overall reliability scores across all questions and in the etiology/clinical presentation domain (p < 0.001) | Prioritize Gemini-2.5-Flash when factual accuracy is paramount (e.g., surgical decision-making discussions) |
| Readability | DeepSeek-V3 | Highest Flesch Reading Ease scores; lowest grade-level indices; shorter, more accessible responses | Use DeepSeek-V3 for patients with lower health literacy or when plain language is essential |
| Reliability–Readability trade-off | All models | No single model achieved both high reliability and recommended readability (6th–8th grade). Gemini responses required 11th–13th grade reading level | Clinician review and simplification of AI-generated content is necessary before sharing with patients |
| Usefulness & global quality | No significant difference | Overall usefulness (GQS) scores did not differ significantly among models (GQS means: ChatGPT 4.16, Gemini 4.16, DeepSeek 4.10 out of 5) | Patients may perceive all models as similarly helpful; clinician oversight is still required regardless of model choice |
| Risk of misleading content | All models | Overly optimistic descriptions of conservative treatment efficacy; inconsistent postoperative recovery timelines; limited discussion of recurrence risk identified | Explicitly correct AI-generated content on orthotics (symptomatic relief only, no corrective effect) and realistic surgical outcomes |
| Clinical integration | All models | AI tools performed variably across clinical domains; no model was uniformly superior across all question categories | AI should function as an adjunct to—not a replacement for—clinician-led patient education, particularly for surgical indications and postoperative expectations |
